# Identification of senescence‐related genes in Parkinson's disease reveals candidate therapeutic targets and pathological processes

**DOI:** 10.1002/ame2.70240

**Published:** 2026-06-30

**Authors:** Haojie Wu, Tingting Liu, Xiangshu Cheng, Jianshe Wei

**Affiliations:** ^1^ Institute for Brain Sciences Research, Center for Translational Neuromedicine, School of Life Sciences Henan University Kaifeng China

**Keywords:** *IRF7*, neuronal dysfunction, NF‐κB signaling pathways, Parkinson's disease, senescence‐related gene

## Abstract

**Background:**

Parkinson's disease (PD), a progressive neurodegenerative disease, is closely linked to the aging process; however, its precise molecular mechanisms remain unclear. This work aimed at clarifying the function of senescence‐related genes (SRGs) in PD and investigating their molecular underpinnings, as well as their potential diagnostic and therapeutic significance.

**Methods:**

By integrating bioinformatics with experimental validation, the CellAge database provided 278 SRGs for this study. A total of 2925 differentially expressed genes (DEGs) and 31 differentially expressed SRGs (DESRGs) were identified, which are mainly involved in signaling pathways, including nuclear factor kappa light chain enhancer of activated B cells (NF‐κB) and interleukin 17 (IL‐17). Through protein–protein interaction (PPI) network analysis, 10 hub genes were identified, such as *IRF7*, *IFNG*, and *BCL6*.

**Results:**

In PD mouse and MN9D cell models by D‐galactose (D‐gal) and 1‐methyl‐4‐phenyl‐1,2,3,6‐tetrahydropyridine (MPTP), combined treatment (D‐gal + MPTP) synergistically reduced tyrosine hydroxylase (TH) expression, promoted α‐synuclein (α‐syn) aggregation, aggravated cellular senescence, and caused significant upregulation of *IRF7*, whereas *IRF7* small interfering RNA (siRNA) intervention reversed senescence‐ and PD‐related pathological changes. Molecular docking analyses identified candidate small molecules targeting hub genes, including fostamatinib and ropsacitinib. In vitro experiments provided preliminary support that these compounds modulated SYK and TYK2 proteins and alleviated pathological phenotypes in cellular models.

**Conclusions:**

Overall, this study demonstrates that D‐gal‐induced aging‐like stress accelerates PD‐related pathological progression by activating multiple signaling pathways, including JAK–STAT, AGE–RAGE, and NF‐κB, thereby promoting inflammation, oxidative stress, and neuronal dysfunction. Key genes including *IRF7* may serve as candidate biomarkers, providing preliminary evidence for the role of senescence‐like stress in PD pathogenesis.

## Graphical abstract

At the genomic level, a large number of differentially expressed genes (DEGs) and aging‐related DEGs have been screened. Ten hub genes, such as IFNγ and IRF7, have been identified and shown potential value in the diagnosis of PD, holding promise as novel biomarkers to facilitate early and precise diagnosis. From the perspective of signaling pathways, multiple pathways, including JAK–STAT, AGE–RAGE, HIF‐1, IL‐17, NF‐κB, and TNF, are activated during the progression of PD and are interwoven with each other. These pathways interfere with the normal function of neurons, disrupt neuronal homeostasis, and promote the development of PD through mechanisms such as inflammation, oxidative stress, and immune responses. Additionally, changes in the expression of related factors during aging interact with the pathological processes of PD, highlighting the crucial role of aging in the pathogenesis of PD.

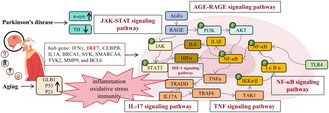

## 1 INTRODUCTION

Among neurodegenerative diseases, Parkinson's disease (PD) is a highly prevalent disorder and is increasingly emerging as a key threat to the health of the global population, particularly among middle‐aged and older adult populations.^1^ After disease onset, patients develop not only motor dysfunction—marked by typical symptoms like tremor, muscle rigidity, and bradykinesia—but also nonmotor manifestations, including cognitive deficits and autonomic nervous system dysfunction.^2^ These symptoms severely disrupt the daily lives of patients by limiting mobility, hindering self‐care, and substantially reducing quality of life. At the same time, the families of these patients are required to devote considerable time and effort to caregiving, which imposes substantial physical, psychological, and economic burdens. From a broader societal perspective, the increasing number of patients with PD has led to greater consumption of medical resources, significantly impacting healthcare systems and economic development.^3^, ^4^

In recent years, global population aging has continued to accelerate. According to authoritative data, the incidence of PD among individuals aged 65 years and older has increased markedly in countries and regions with advanced aging demographics.^5^ As humans age, the body—much like a gradually aging precision machine—experiences a progressive decline in the performance of its various components, and the nervous system is no exception. During aging, neuronal function deteriorates, information transmission efficiency decreases, and the ability to regulate bodily functions weakens. Simultaneously, intracellular homeostasis becomes disrupted, leading to disturbances in previously well‐regulated processes such as cellular metabolism and material transport.^6^ These age‐related changes closely mirror the pathological features of PD. For example, declining neuronal function is strongly associated with motor and intellectual impairments in PD patients, whereas disrupted intracellular homeostasis may promote the accumulation of toxic substances within cells, further exacerbating neuronal damage.^7^, ^8^ Together, these observations strongly suggest a complex and intrinsic link between aging and PD.

Although previous studies have demonstrated a close association between aging and PD, and multiple inflammatory, oxidative stress, and signaling pathways are believed to be involved, existing research still lacks systematic screening and validation of senescence‐related genes (SRGs) in PD, particularly multilevel experimental evidence ranging from bioinformatics analysis to animal and cellular models.^9^, ^10^ Furthermore, the molecular mechanisms by which aging regulates the pathological progression of PD through specific key genes remain unclear, and stable models and key regulatory targets that can simultaneously reflect aging stress and PD‐like pathology still need to be further identified. Additionally, existing research has not sufficiently explored potential diagnostic biomarkers and candidate intervention targets for aging‐related PD, limiting the development of early disease identification and precision interventions.

Through sustained efforts by researchers over recent decades, significant progress has been made in understanding the relationship between PD and aging.^11^, ^12^, ^13^ Building on this foundation, the uniqueness of this study lies in the following: for the first time, we systematically integrated public transcriptomic data with aging‐related genes to identify differentially expressed aging‐related genes in PD and construct a regulatory network; we simultaneously used a d‐galactose (D‐gal) combined with 1‐methyl‐4‐phenyl‐1,2,3,6‐tetrahydropyridine (MPTP)/MPP^+^ to establish a PD model mimicking aging‐related stress, thereby validating the roles of key genes and signaling pathways at both the animal and cellular levels; and we preliminarily explored potential small‐molecule interventions targeting hub genes, providing a more comprehensive evidence chain for understanding the aging‐related pathological mechanisms of PD.

This study focuses on the intrinsic link between aging‐related genes and PD. First, bioinformatics analyses were conducted to identify differentially expressed SRGs (DESRGs). Subsequently, functional enrichment analysis was performed to determine the roles of these genes in biological processes (BP), cellular components (CC), and molecular functions (MF). Meanwhile, a protein–protein interaction (PPI) network was constructed, through which key hub regulatory genes were identified. To validate the potential roles of these genes and signaling pathways in the pathogenesis and progression of PD, we established relevant animal and cellular models. In animal studies, PD‐like pathological models, d‐gal‐induced aging stress models, and combined pathological models were established, and behavioral deficits and pathological alterations were evaluated to determine the functional relevance of these genes and pathways at the whole‐animal level. In cellular experiments, we investigated the underlying mechanisms by performing in‐depth analyses of physiological changes and gene expression at the cellular and molecular levels. Through comprehensive validation using mouse models and the MN9D cell line, we preliminarily characterized the molecular mechanisms linking d‐gal‐induced aging‐like stress to PD‐related pathology.

Through these systematic investigations, the present study explores the potential roles of d‐gal‐induced aging‐like stress in PD‐related pathogenesis. These findings provide preliminary insights into the molecular mechanisms underlying senescence‐associated PD‐like pathology, which may offer potential clues for further mechanistic studies and future drug development.

## 2 MATERIALS AND METHODS

### 2.1 Data acquisition

We extracted 278 SRGs in total from the CellAge database, with the complete list provided in Table S1. In addition, two microarray expression datasets (GSE20141 and GSE7621) were downloaded from the Gene Expression Omnibus (GEO) database. Both datasets were generated on the GPL570 platform and included the following samples: GSE20141 contained 10 PD patients and 8 healthy controls, whereas GSE7621 contained 16 PD patients and 9 healthy controls. The GSE48350 and GSE5281 datasets were also downloaded from the GEO database using the GEOquery package. We removed batch effects across datasets (treated as independent batches) using the ComBat function in the sva package. Probes matching multiple genes were excluded; for genes represented by multiple probes, only the probe with the strongest signal intensity was retained. Principal component analysis (PCA) was performed to evaluate sample distribution patterns and the efficacy of batch correction. The limma package was used to identify differentially expressed genes (DEGs) between distinct groups. The results were visualized using volcano plots, and significantly DEGs were presented in heatmaps. All analyses were performed using R software (version 4.2.1), with the main R packages including GEOquery (version 2.64.2), limma (version 3.52.2), ggplot2 (version 3.4.4), and ComplexHeatmap (version 2.13.1).^14^, ^15^, ^16^

### 2.2 Differential gene analysis

The intersection between DEGs and SRGs, defined as DESRGs, was identified and visualized using the venneuler package in R. Functional enrichment analyses of DEGs were performed using the clusterProfiler and GOplot packages in R to evaluate the associated Kyoto Encyclopedia of Genes and Genomes (KEGG) pathways and Gene Ontology (GO) terms. The STRING database was used to perform PPI analysis of DESRGs.^17^ Subsequently, the 10 most significant hub genes were identified using the maximal clique centrality (MCC) algorithm implemented in the CytoHubba plugin of Cytoscape (version 3.9.0). The biological functions of these hub genes were annotated using the GeneCards database (https://www.genecards.org/).^18^ Receiver operating characteristic (ROC) curve analysis was performed to explore the candidate diagnostic value of hub genes, and the results were visualized. To further assess the performance of these hub genes, the area under the ROC curve (AUC) was calculated using the pROC package in R; genes with an AUC >0.70 were provisionally considered as potential candidate diagnostic indicators.

### 2.3 Experiment validation

2.3.1

**FIGURE 1:**
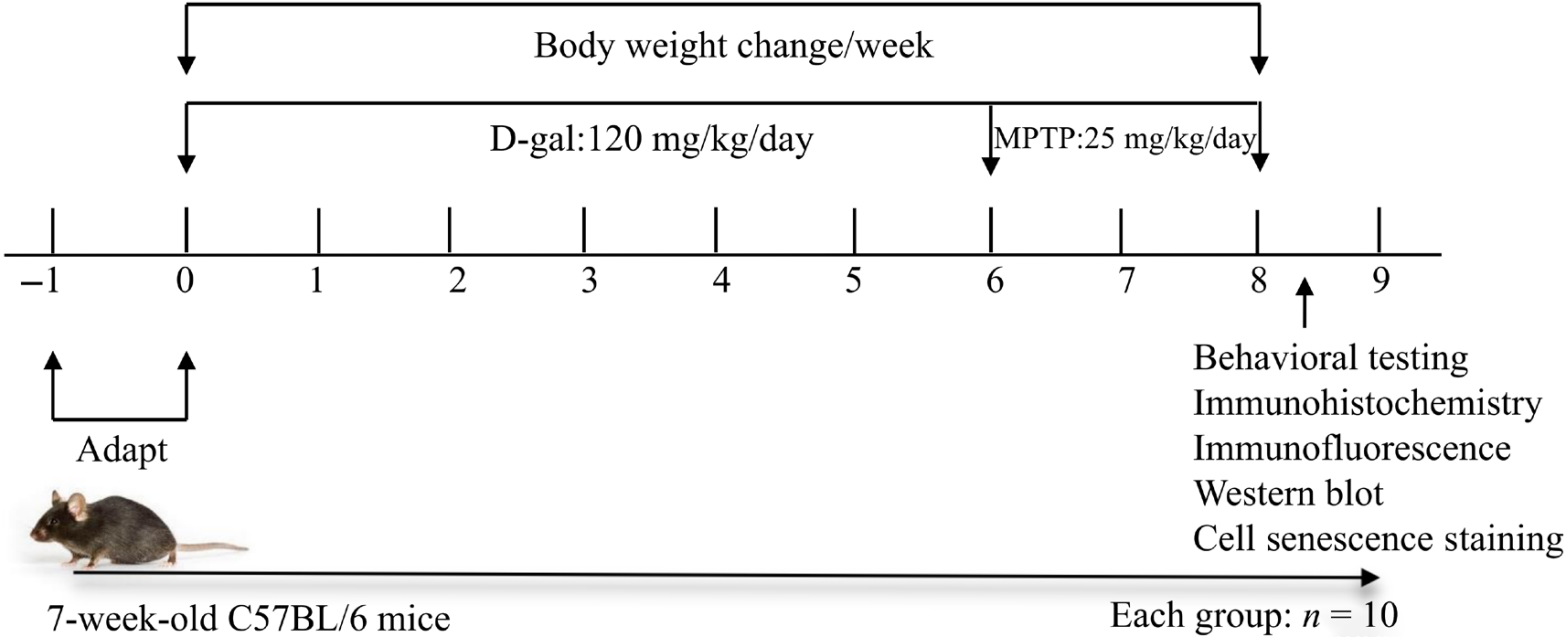
The timeline of 1‐methyl‐4‐phenyl‐1,2,3,6‐tetrahydropyridine (MPTP)‐induced Parkinson's disease (PD) mice model in vivo experiments.

We obtained male C57BL/6J mice (7‐week‐old; 20–22 g) from Cyagen Biosciences. All animals were housed in a controlled environment (22 ± 2°C, 55% ± 5% humidity, 12/12‐h light/dark cycle) and given ad libitum access to food and water. All experimental protocols involving animals were approved by the Animal Care and Use Committee of Henan University (approval no.: HUSOM2021‐161), and all animal experiments were performed in strict accordance with the institutional guidelines for laboratory animal care and use and the ARRIVE 2.0 guidelines. After a 1‐week acclimation period, mice were randomly assigned to four groups (*n* = 10 per group): (1) saline group, (2) d‐gal group (Abmole, M9976; 120 mg/kg/day, intraperitoneal injection for 6 weeks),^19^ (3) MPTP group (Abmole, M9049; 20 mg/kg/day for 2 weeks),^20^ and (4) d‐gal + MPTP group (d‐gal for 6 weeks followed by MPTP for 2 weeks). Mice in the saline group received an equivalent volume of normal saline via intraperitoneal injection daily for 8 weeks. Behavioral tests were performed at 3:00 p.m. after the end of the treatment period, and all tests were conducted in a single‐blinded manner, with testers and data analysts blinded to the group allocation of mice throughout the process. Subsequently, five mice from each group were randomly selected for transcardial perfusion with 0.9% saline followed by 4% paraformaldehyde fixation, after which brain tissues were dissected for subsequent histological analysis; all histological image acquisition and quantitative analysis were also performed in a single‐blinded manner by two independent researchers. The remaining five mice per group were euthanized, and substantia nigra (SN) tissues were immediately dissected on ice, snap‐frozen in liquid nitrogen, and stored at −80°C for subsequent experiments. The experimental timeline is shown in Figure 1.

### 2.4 Behavioral testing

#### 2.4.1 Gait test

A gray acrylic runway (3 mm thickness; 200 × 20 × 12 cm) was used for gait analysis. Mice were acclimated to the runway on the first day. Step length was defined as the average distance between forepaw and hindpaw prints as the mouse moved along the runway. At least six stride measurements were collected per mouse for analysis.

#### 2.4.2 Rotarod test

Mice were placed in individual lanes on a rotarod apparatus. After 300 s at 4 rpm, the speed was increased to 40 rpm and maintained for 300 s. The latency to fall was recorded as the average time each mouse remained balanced on the rotating rod.

#### 2.4.3 Tail suspension test

Mice were hung by their tails using a thin string 50 cm above the floor to assess depressive‐like behavior. The duration of immobility (defined as no limb or body movement) was recorded during the test period.

#### 2.4.4 Forced swimming

For the forced swimming test, mice from each group were individually placed in a cylindrical container (10 cm diameter, 25 cm height) filled with 19 mL of distilled water maintained at 25 ± 1°C. Immobility time during the 5‐min test was measured as an indicator of depression‐like behavior.

#### 2.4.5 Open‐field test

The open‐field apparatus consisted of a square arena (50 × 50 × 30 cm) illuminated by a 100‐Lx light source positioned 150 cm above the center. Mice were placed in a 15 × 15 cm central zone, and their locomotor activity was recorded for 5 min, including velocity, total distance traveled, and time spent at the center.

#### 2.4.6 Elevated plus maze test

An elevated plus maze with two open arms (50 × 10 cm), two closed arms (50 × 10 × 35 cm), and a central platform (10 × 10 cm) was employed, with the apparatus positioned 50 cm above the ground. Each mouse was placed on the central platform, facing an open arm, and allowed to explore the maze for 5 min. The time spent in open and closed arms was measured, and the proportion of open‐arm occupancy relative to total exploration time (all arms combined) was calculated.

### 2.5 Cell culture

The MN9D dopaminergic neuronal cell line was obtained from Servicebio (Wuhan, China). Cells were cultured in high‐glucose Dulbecco's modified Eagle medium (DMEM, Gibco) supplemented with 10% fetal bovine serum, 100 U/mL penicillin, and 100 U/mL streptomycin. Cells were maintained in a humidified incubator at 37°C with 5% CO_2_. The culture medium was refreshed every 2–3 days to maintain cell viability. Cell was divided into groups based on d‐gal concentrations (MCE; HY‐N0210): 0 (control), 20, 40, 60, and 80 mg/mL for subsequent experiments. Additional groups included d‐gal (40 mg/mL), MPP^+^ (Abmole; M10041; 100 μmol/L), and D‐gal + MPP^+^. These groupings allowed a complete study of the effects of d‐gal and MPP^+^ on MN9D cells. To evaluate the role of *IRF7* in aging‐related PD, cells were divided into control, D‐gal, MPP^+^, d‐gal + MPP^+^, and d‐gal + MPP^+^ + *IRF7* small interfering RNA (siRNA) (MCE; HY‐RS16952; 5 nmol/L) groups.

### 2.6 Cell viability assay

Cells were plated in 96‐well plates at 100 μL per well and treated with various concentrations of d‐gal (0, 20, 40, 60, and 80 mg/mL). After incubation at 37°C with 5% CO_2_ for an appropriate period, 10 μL of CCK‐8 solution (Servicebio; G4103) was added to each well, and plates were incubated for a further 2 h. Cell viability was measured at 450‐nm absorbance using a microplate reader.

### 2.7 Western blot

SN tissues were homogenized in ice‐cold radioimmunoprecipitation assay buffer containing phenylmethylsulfonyl fluoride (Servicebio, G2002, G2008) using a Polytron homogenizer. The homogenates were sonicated and then centrifuged at 12 000 × *g* for 10 min at 4°C to remove debris. Protein concentrations in the supernatants were determined using a bicinchoninic acid (BCA) protein assay kit. Equal amounts of protein were separated by sodium dodecyl sulfate‐polyacrylamide gel electrophoresis (SDS‐PAGE) and transferred to a nitrocellulose membrane (Millipore, IPFL00010, Germany). After transfer, membranes were blocked in 5% nonfat milk in Tris‐Buffered Saline with Tween‐20 (TBST) at 37°C for 1 h, and then washed twice with TBST. Membranes were incubated overnight at 4°C with primary antibodies diluted in TBST containing 1% nonfat milk. After three washes with TBST, membranes were incubated with horseradish peroxidase (HRP)‐conjugated secondary antibodies (diluted in TBST containing 1% nonfat milk) for 2 h at room temperature. Protein bands were visualized using an enhanced chemiluminescence detection system (Bio‐Rad).

### 2.8 Immunohistochemistry

Immunohistochemical staining was performed on 25‐μm‐thick serial SN brain sections. Sections were heated at 98°C for 10 min in 1× citrate antigen retrieval buffer, treated with 0.1% Triton X‐100 for 10 min to permeabilize cells, and then incubated with 3% hydrogen peroxide in the dark for 20 min to quench endogenous peroxidase activity. After being blocked with 10% goat serum in phosphate‐buffered saline (PBS) for 30 min at room temperature, sections were incubated with the appropriate primary antibodies at 4°C overnight. Sections were then washed with PBS and incubated with biotin‐conjugated secondary antibodies. Immunoreactivity was visualized using a DAB chromogenic substrate kit (Servicebio, G1212). All image acquisition and quantitative analysis for this assay were performed in a single‐blinded manner, with analysts blinded to the group allocation of samples throughout the process. Images were captured using a Leica DMI4000B microscope (Wetzlar, Germany) and analyzed using ImageJ software. Quantitative analysis was performed based on optical density measurements.

### 2.9 Immunofluorescence

Immunofluorescence analysis was performed on 30‐μm‐thick serial brain sections and MN9D cell samples. Samples were subjected to antigen retrieval in 1× citrate buffer at 98°C for 10 min, washed thrice with PBS, permeabilized with 0.1% Triton X‐100 for 10 min, and then washed again thrice with PBS. Sections and cells were blocked with 10% goat serum in PBS for 30 min at room temperature and incubated overnight at 4°C with the corresponding primary antibodies (1:200; Affinity). After three 5‐min washes with PBS, samples were incubated with Alexa Fluor 555/488‐conjugated secondary antibodies (1:2000; Invitrogen) for 2 h at room temperature in the dark, followed by DAPI (4′,6‐diamidino‐2‐phenylindole) nuclear staining for 10 min. Sections were mounted using Vector Laboratories' Vectashield mounting medium. All image acquisition and quantitative analysis for this assay were performed in a single‐blinded manner, with analysts blinded to the group allocation of samples throughout the process. Images were captured using a Leica DMI4000B fluorescence microscope. The mean fluorescence intensity (MFI) of target proteins was quantified using ImageJ.

### 2.10 Cell senescence staining and reactive oxygen species detection

Cell senescence and reactive oxygen species (ROS) levels were analyzed in 30‐μm‐thick brain sections and MN9D cell cultures using a senescence GLB1 (SA‐β‐gal) staining kit (Beyotime, C0602), Senescence‐Tracker fluorescent probe (Beyotime, C0603), and ROS fluorescent probe (Servicebio, G1706).

### 2.11 Potential therapeutic drug prediction

To identify potential therapeutic targets for PD, the DGIdb database was used to construct a gene–drug interaction network.^21^ In silico protein–ligand docking was performed using AutoDock Vina (version 1.2.2) to evaluate the binding affinities and interaction modes between candidate drugs and target proteins. Compound structures were retrieved from the PubChem database,^22^ and three‐dimensional protein structures were downloaded from the PDB.^23^ Protein and ligand files were converted to PDBQT format, water molecules were removed, polar hydrogen atoms were added, and grid boxes were placed on the functional domains of the target protein to allow free movement of the ligand. The grid box dimensions were set to 30 × 30 × 30 Å with a grid spacing of 0.05 nm.^24^ Compounds with lower binding energies in the molecular docking analysis of drugs and proteins were selected for experimental validation, including fostamatinib (2 μmol/L; MCE, HY‐13038), ropsacitinib (10 μmol/L; Proteintech, CM04910), and MK‐5108 (1 μmol/L; MCE, HY‐13252). The therapeutic effects of these compounds were subsequently evaluated in cell‐based models.

### 2.12 Statistical analysis

All statistical analyses were performed using GraphPad Prism 9.0, with data presented as mean ± standard deviation (SD) and a two‐sided significance threshold of *p* < 0.05. Two‐group comparisons used an unpaired Student's *t*‐test; three or more group comparisons used one‐way analysis of variance (ANOVA) with Tukey's honestly significant difference (HSD) test (all pairwise comparisons) or Dunnett's (multiple groups vs. single control) post‐hoc test, with Bonferroni's correction for multiple comparisons. The “*n*” value indicates biological replicates (independent animals for in vivo work, independent cell experiments for in vitro work). Behavioral testing and histologic image acquisition and quantification were single blinded by two independent researchers blinded to group allocation. ROC curve analysis with AUC and 95% confidence interval (CI) calculation was used to assess gene diagnostic performance; AUC >0.70 was set as the candidate gene inclusion threshold, consistent with standard practice for exploratory biomarker discovery.

## 3 RESULTS

### 3.1 DEG acquisition and analysis

Data normalization was performed on datasets GSE20141 and GSE7621, which included 26 PD patients and 17 healthy control samples. DEGs were screened using the following criteria: corrected *p*‐value <0.05 and |logFC| >0.58. PCA and boxplot distribution patterns jointly confirmed the reproducibility and reliability of the normalized data. Volcano plots were used to visualize DEGs, and 2925 DEGs were ultimately identified in the integrated PD dataset (Figure S1).

### 3.2 Biological function enrichment analysis

**FIGURE 2:**
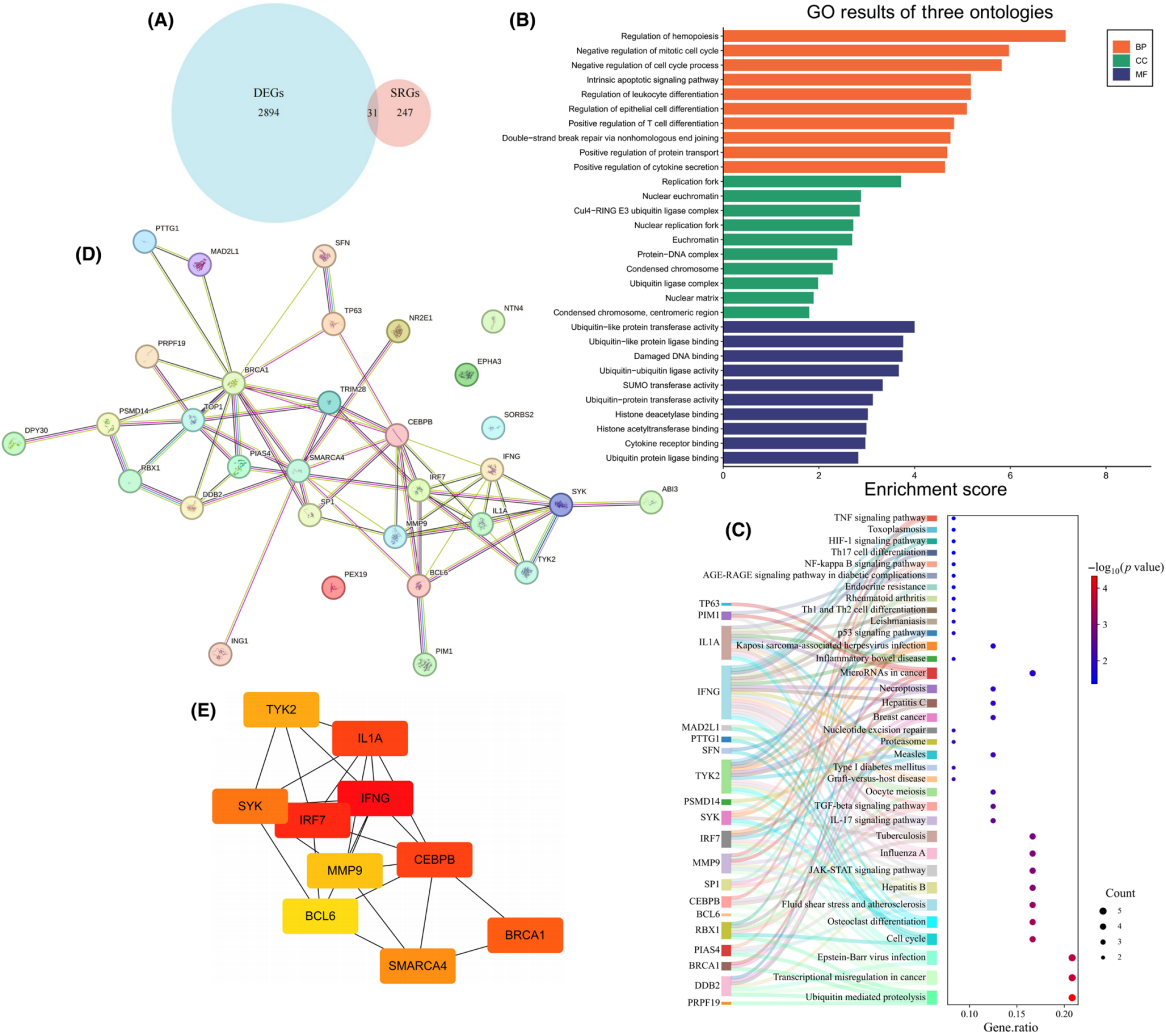
Differential gene analysis. (A) Venn diagram of overlapping genes between senescence‐related genes (SRGs) and differentially expressed genes (DEGs). Biological function and Kyoto Encyclopedia of Genes and Genomes (KEGG) enrichment analysis, including (B) Gene Ontology Biological Process (GOBP), Gene Ontology Cellular Component (GOCC), Gene Ontology Molecular Function (GOMF), and (C) pathway. (D) Protein–protein interaction (PPI) analysis of differentially expressed SRGs (DESRGs). (E) Maximal clique centrality (MCC) algorithm analyzes hub genes.

The intersection of 278 SRGs and DEGs yielded 31 DESRGs (Figure 2A). Biological functional enrichment analysis was performed across three GO categories: BP, CC, and MF (Figure 2B). In the BP category, DESRGs were mainly enriched in hematopoiesis regulation, mitotic cell cycle, negative regulation of cell cycle processes, endogenous apoptotic signaling pathways, leukocyte differentiation, and epithelial cell differentiation. In the CC category, enrichment was observed in the replication fork, nuclear euchromatin, Cul4‐RING E3 ubiquitin ligase complex, nuclear replication fork, euchromatin, protein–DNA complexes, condensed chromosomes, ubiquitin ligase complexes, nuclear matrix, and centromeric regions. In the MF category, DESRGs were predominantly enriched in ubiquitin‐like protein transferase activity, ubiquitin‐like protein ligase binding, damaged DNA binding, ubiquitin–ubiquitin ligase activity, SUMO transferase activity, ubiquitin–protein transferase activity, and histone deacetylase binding. KEGG pathway analysis showed that DEGs were strongly correlated with the JAK–STAT, interleukin 17 (IL‐17), and tumor growth factor β (TGF‐β) signaling pathways. We also identified pathways related to endocrine resistance, the diabetic complication AGE–RAGE signaling pathway, nuclear factor kappa light chain enhancer of activated B cells (NF‐κB) signaling, T helper 17 (Th17) cell differentiation, hypoxia‐inducible factor 1 (HIF‐1) signaling, toxoplasmosis, and tumor necrosis factor (TNF) signaling (Figure 2C).

### 3.3 PPI network and hub gene analysis

PPI analysis using STRING identified a 31‐node, 62‐edge network, indicating a high level of connectivity. The average node degree was 4, suggesting moderate interaction density, whereas the average local clustering coefficient was 0.593, indicating a strong tendency for local community formation. Based on random model predictions, the expected number of edges was 31; however, the observed number of edges was 62, far exceeding expectations. Furthermore, the PPI enrichment *p*‐value was 4.22 × 10^−7^, strongly indicating that the observed interactions were not random but significantly enriched, thereby underscoring the biological relevance of this network (Figure 2D). Hub genes identified using the MCC algorithm included *IFNγ*, *IL1α*, *IRF7*, *MMP9*, *TYK2*, *BCL6*, *SYK*, *CEBPβ*, *BRCA1*, and *SMARCA4* (Figure 2E). The biological functions of these hub genes are summarized in Table S2.

### 3.4 In vivo experiments

#### 3.4.1 Behavioral tests

**FIGURE 3:**
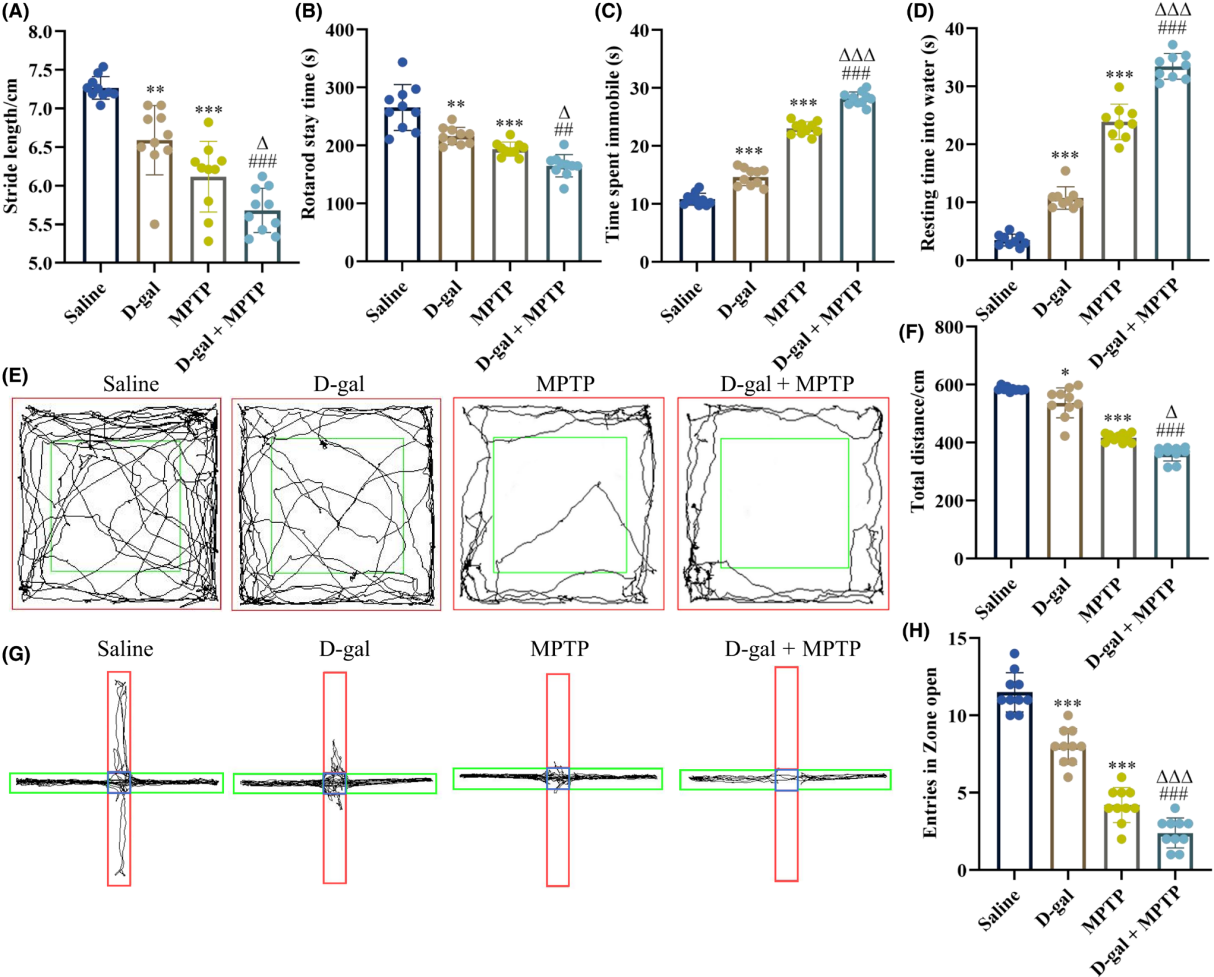
Behavioral tests. (A) Gait test (stride length, in centimeters). (B) Rotarod test (retention time, in seconds). (C) Tail suspension test (immobility time, in seconds). (D) Forced swimming test (immobility time, in seconds). (E) Representative trajectories of the open‐field test. (F) Total travel distance of open‐field test (in centimeters). (G) Representative trajectories of the elevated plus maze test. (H) Number of open arm entries in the elevated plus maze test. *n* = 10 per group; data are presented as mean ± standard deviation (SD). One‐way analysis of variance (ANOVA) with Tukey's honestly significant difference (HSD) post‐hoc test and Bonferroni's correction. *p < 0.05, **p < 0.01, ***p < 0.001 versus saline; ^##^ *p* < 0.01, ^###^ *p* < 0.001 versus d‐gal; ^∆∆^ *p* < 0.01, ^∆∆∆^ *p* < 0.001 versus 1‐methyl‐4‐phenyl‐1,2,3,6‐tetrahydropyridine (MPTP).

Compared to the saline group, both d‐gal and MPTP treatment induced motor deficits, as evidenced by the reduced step length in the d‐gal and MPTP groups (Figure 3A). These groups also exhibited significantly shorter fall times in the rotarod test (Figure 3B). Depression‐like behaviors induced by both interventions were verified using forced swim and tail suspension tests, with a marked increase in immobility time observed in the MPTP group (Figure 3C,D). Additionally, d‐gal and MPTP exposure induced stress‐related behaviors, reflected by a reduction in total distance traveled in the open‐field test (Figure 3E,F). Through the elevated plus maze experiment, we found that d‐gal and MPTP mice spent less time on the open arms (Figure 3G,H). Notably, the d‐gal + MPTP group exhibited synergistic effects, showing more severe motor impairments, depression‐like behaviors, and anxiety compared to either treatment alone. Analysis of body weight changes over 8 weeks showed significant weight gain in the saline group. It was found that the rate of weight gain in the d‐gal + MPTP group was significantly slower (Figure S2A).

#### 3.4.2 Pathological assessment of the SN

**FIGURE 4:**
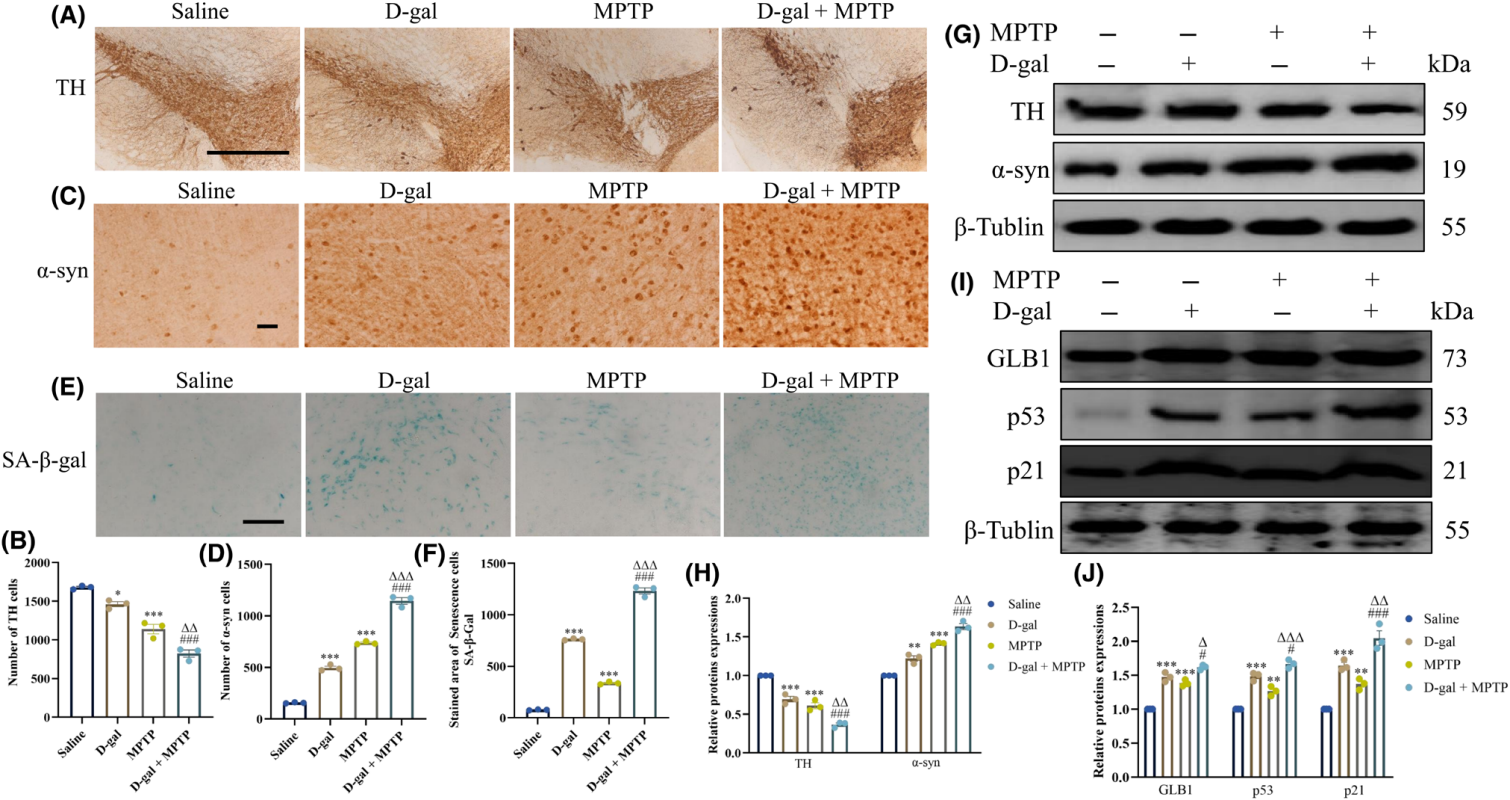
Pathology index detection. (A) Tyrosine hydroxylase (TH) immunohistochemistry in the substantia nigra (SN) (scale bar = 100 μm). (B) Quantification of TH‐positive dopaminergic neurons. (C) α‐Synuclein (α‐syn) immunohistochemistry in the SN (scale bar = 20 μm). (D) Quantification of α‐syn‐positive cells. (E) SA‐β‐gal senescence staining in the SN (scale bar = 50 μm). (F) Quantification of SA‐β‐gal–positive staining area. (G) Western blot of TH and α‐syn (β‐tubulin as internal control). (H) Relative protein levels of TH and α‐syn. (I) Western blot of senescence‐related proteins GLB1, p53, and p21 (β‐tubulin as internal control). (J) Relative protein levels of GLB1, p53, and p21. *n* = 3 biological replicates per group; data are presented as mean ± standard deviation (SD). One‐way analysis of variance (ANOVA) with Tukey's honestly significant difference (HSD) post‐hoc test and Bonferroni's correction. *p < 0.05, **p < 0.01, ***p < 0.001 versus saline; ^#^ *p* < 0.05, ^###^ *p* < 0.001 versus ^∆^ *p* < 0.05, ^∆∆^ *p* < 0.01, ^∆∆∆^ *p* < 0.001 versus 1‐methyl‐4‐phenyl‐1,2,3,6‐tetrahydropyridine (MPTP).

In the saline group, tyrosine hydroxylase (TH) staining was intense and densely distributed. In contrast, TH staining intensity and coverage were reduced in the d‐gal and MPTP groups. Moreover, staining was further diminished in the combined treatment group, indicating that both interventions reduced TH expression, with a more pronounced effect following co‐treatment (Figure 4A,B). Immunofluorescence analysis yielded consistent results (Figure S2B,C). α‐Synuclein (α‐syn) staining was faint and sparse in the saline group but gradually increased in intensity and distribution in the d‐gal, MPTP, and d‐gal + MPTP groups, suggesting that both treatments promoted α‐syn aggregation, with the strongest effect observed under combined administration (Figure 4C,D). SA‐β‐gal staining was nearly absent in the saline group but progressively increased in the positive area in the d‐gal, MPTP, and d‐gal + MPTP groups, indicating the induction of cellular senescence, particularly by co‐treatment (Figure 4E,F). TH protein expression was highest in the saline group, moderately decreased in the d‐gal and MPTP groups, and lowest in the d‐gal + MPTP group. Conversely, α‐syn expression was gradually upregulated across the d‐gal, MPTP, and d‐gal + MPTP groups (Figure 4G,H). The senescence‐associated proteins GLB1, p53, and p21 also exhibited progressively elevated expression in these groups (Figure 4I,J).

#### 3.4.3 Hub gene analysis

**FIGURE 5:**
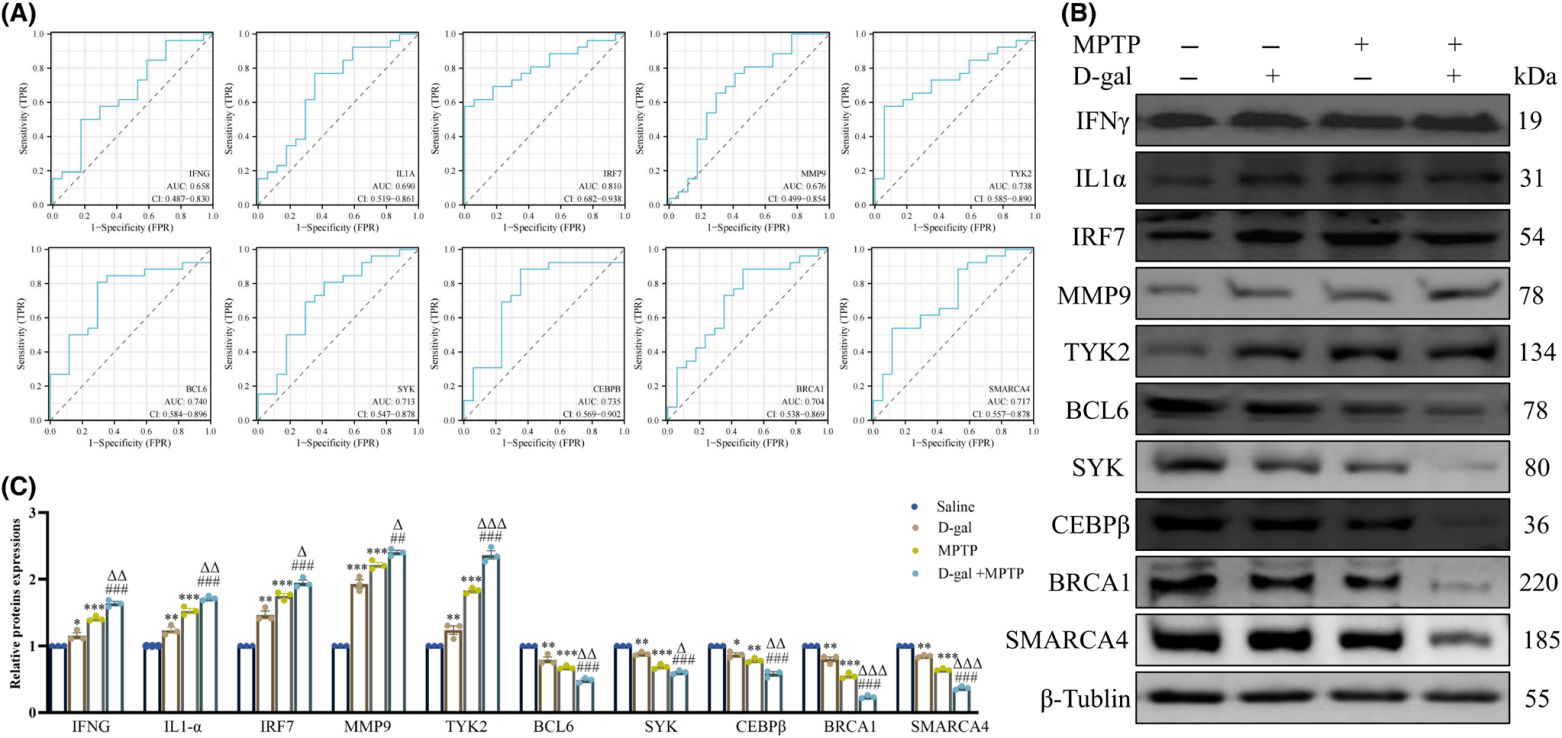
Hub gene analysis. (A) Receiver operating characteristic (ROC) curve analysis of the diagnostic performance of 10 hub genes for Parkinson's disease (PD) in the integrated Gene Expression Omnibus (GEO) datasets (area under the ROC curve [AUC] and 95% confidence interval [CI] labeled for each gene). (B) Western blot of 10 hub proteins (IFNγ, IL1α, IRF7, MMP9, TYK2, BCL6, SYK, CEBPβ, BRCA1, SMARCA4) in mouse substantia nigra (SN) tissues (β‐tubulin as internal control). (C) Quantification of the relative protein levels of the 10 hub genes, normalized to β‐tubulin. *n* = 3 biological replicates per group, data are presented as mean ± standard deviation (SD). One‐way analysis of variance (ANOVA) with Tukey's honestly significant difference (HSD) post‐hoc test and Bonferroni's correction. *p < 0.05, **p < 0.01, ***p < 0.001 versus saline; ^##^ *p* < 0.01, ^###^ *p* < 0.001 versus d‐gal; ^∆^ *p* < 0.05, ^∆∆^ *p* < 0.01, ^∆∆∆^ *p* < 0.001 versus 1‐methyl‐4‐phenyl‐1,2,3,6‐tetrahydropyridine (MPTP).

We evaluated the diagnostic efficacy of the 10 hub genes for PD using ROC curve analysis. The AUC values for these genes in the PD dataset were as follows: *IFNG* (0.658; CI: 0.487–0.830), *IL1α* (0.690; CI: 0.519–0.861), *IRF7* (0.810; CI: 0.682–0.938), *MMP9* (0.676; CI: 0.499–0.854), *TYK2* (0.738; CI: 0.585–0.890), *BCL6* (0.740; CI: 0.584–0.896), *SYK* (0.713; CI: 0.547–0.878), *CEBPβ* (0.735; CI: 0.569–0.902), *BRCA1* (0.704; CI: 0.538–0.869), and *SMARCA4* (0.717; CI: 0.557–0.878). An AUC value closer to 1 indicates stronger discriminatory power between PD patients and controls. Among these genes, *IRF7* exhibited the highest AUC (0.810), demonstrating strong diagnostic potential (Figures 5A and S3A). Further analysis showed that compared to the saline group, the expression levels of IFNG, IL1α, IRF7, MMP9, and TYK2 were elevated in the d‐gal and MPTP groups, whereas BCL6, SYK, CEBPβ, BRCA1, and SMARCA4 were downregulated. These expression changes suggest that the dysregulation of these genes may be associated with PD pathogenesis. Moreover, these expression trends were more pronounced in the d‐gal + MPTP group, indicating that combined treatment exerts a stronger impact on PD‐related gene expression (Figure 5B,C). Immunohistochemical analysis further confirmed that BRCA1 and SMARCA4 expression patterns in the SN were consistent with the protein expression data (Figure S3B–E).

#### 3.4.4 Analysis of related signaling pathways

**FIGURE 6:**
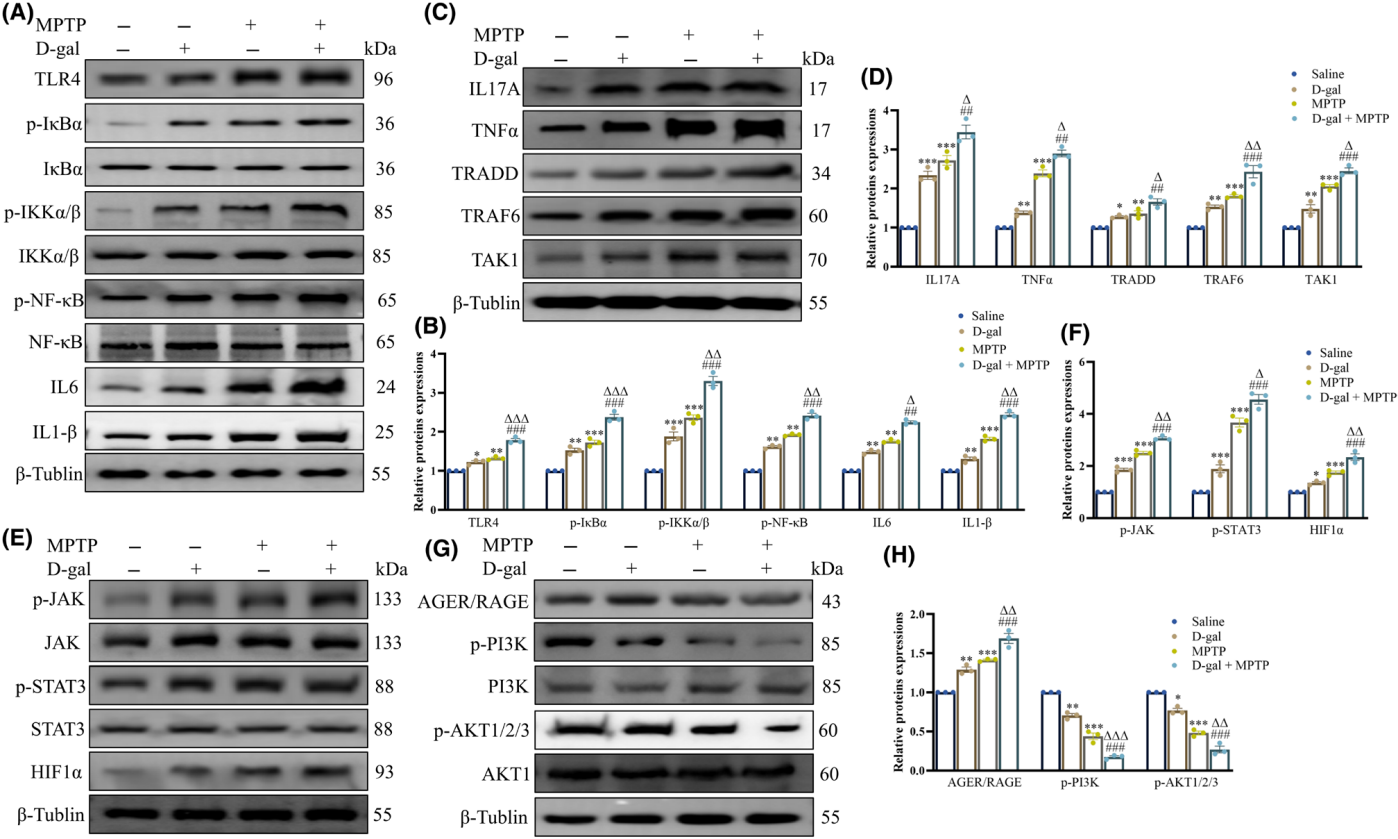
Analysis of related signaling pathways. (A) Western blot of nuclear factor kappa light chain enhancer of activated B cells (NF‐κB) pathway key proteins (TLR4, p‐IκBα, IκBα, p‐IKKα/β, IKKα/β, p‐NF‐κB, NF‐κB, IL6, IL‐1β), with β‐tubulin as internal control. (B) Relative protein quantification of panel A. (C) Western blot of interleukin 17 (IL‐17)/tumor necrosis factor (TNF) pathway key proteins (IL17A, TNF‐α, TRADD, TRAF6, TAK1), with β‐tubulin as internal control. (D) Relative protein quantification of panel C. (E) Western blot of JAK–STAT/HIF‐1 pathway key proteins (p‐JAK, JAK, p‐STAT3, STAT3, HIF1α), with β‐tubulin as internal control. (F) Relative protein quantification of panel E. (G) Western blot of AGE–RAGE pathway key proteins (RAGE, p‐PI3K, PI3K, p‐AKT1/2/3, AKT1), with β‐tubulin as internal control. (H) Relative protein quantification of panel G. *n* = 3 biological replicates per group; data are presented as mean ± standard deviation (SD). One‐way analysis of variance (ANOVA) with Tukey's honestly significant difference (HSD) post‐hoc test and Bonferroni's correction. *p < 0.05, **p < 0.01, ***p < 0.001 versus saline; ^##^ *p* < 0.01, ^###^ *p* < 0.001 versus d‐gal; ^∆^ *p* < 0.05, ^∆∆^ *p* < 0.01, ^∆∆∆^ *p* < 0.001 versus 1‐methyl‐4‐phenyl‐1,2,3,6‐tetrahydropyridine (MPTP).

Pathway enrichment analysis demonstrated the activation of multiple critical signaling cascades. First, within the NF‐κB signaling pathway, d‐gal, MPTP, and their combined treatment (d‐gal + MPTP) activated several downstream molecules, including TLR4, phosphorylated IKKα/β (p‐IKKα/β), phosphorylated NF‐κB (p‐NF‐κB), phosphorylated IκBα (p‐IκBα), IL‐6, and IL‐1β (Figure 6A,B). In the TNF signaling pathway, TNF‐α activated TRADD, TRAF6, and TAK1. In addition, IL‐17α activation was observed in the IL‐17 signaling pathway (Figure 6C,D). Concurrently, phosphorylation of JAK and STAT3 was detected in the JAK–STAT signaling pathways (Figure 6E,F). In the AGE–RAGE signaling pathway, RAGE expression was activated, whereas phosphorylation of PI3K and AKT was inhibited (Figure 6G,H). Finally, HIF1α in the HIF‐1 signaling pathway was activated through interactions with the NF‐κB and JAK–STAT signaling pathways. These complex signaling activation profiles played critical roles in PD pathogenesis and provided insights into disease mechanisms and potential therapeutic strategies.

### 3.5 In vitro experiments

#### 3.5.1 Construction of a cellular senescence model

**FIGURE 7:**
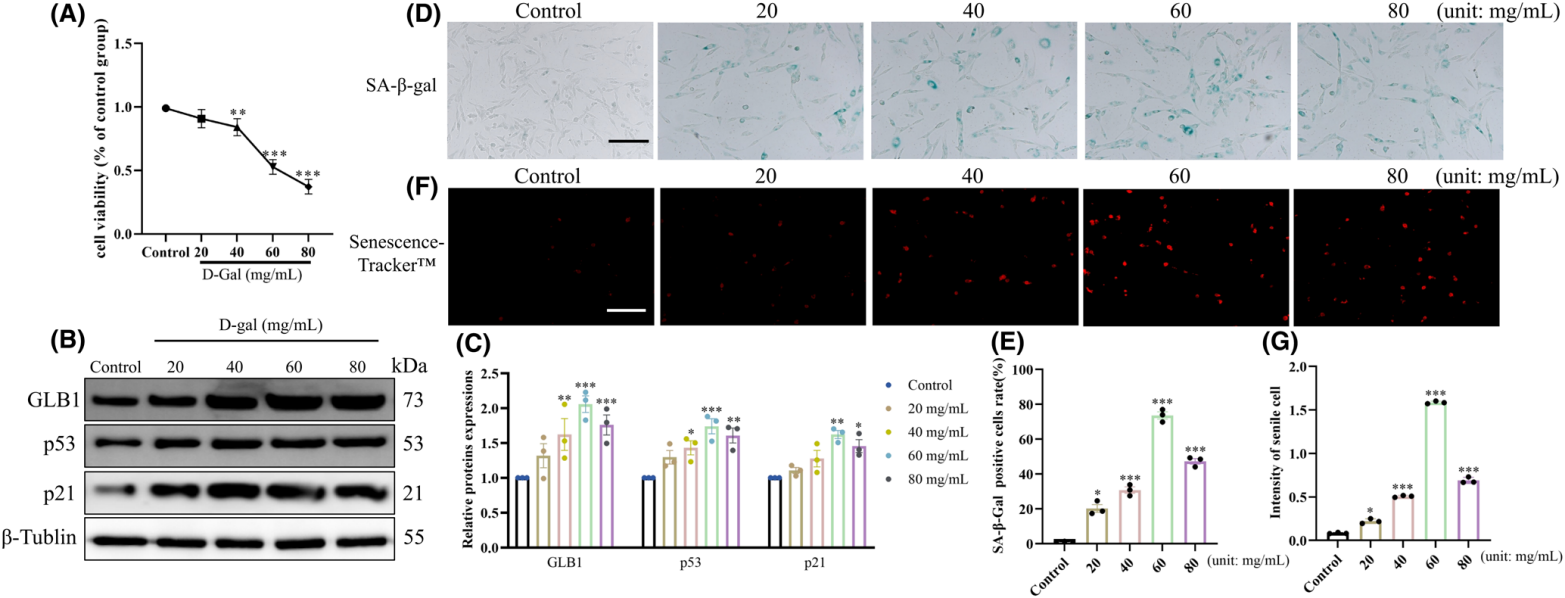
Optimal concentration selection for cellular senescence models. (A) CCK‐8 assay of MN9D cell viability under gradient d‐gal treatment (0, 20, 40, 60, 80 mg/mL) on MN9D cells. *n* = 5. (B) Western blot of senescence‐related proteins GLB1, P53, P21 (β‐tubulin as internal control). (C) Relative protein quantification of panel B. (D) SA‐β‐gal staining of MN9D cells (scale bar = 50 μm); (E) Quantification of SA‐β‐gal–positive cell percentage. (F) Senescence‐Tracker fluorescent probe detection of senescent cells (scale bar = 50 μm). (G) Quantification of relative fluorescence intensity. *n* = 5 biological replicates for panel A, *n* = 3 for panels B–G. Data are presented as mean ± standard deviation (SD). One‐way analysis of variance (ANOVA) with Dunnett's post‐hoc test and Bonferroni's correction. *p < 0.05, **p < 0.01, ***p < 0.001 versus control group.

Compared to the control group, cell viability decreased significantly in a concentration‐dependent manner as d‐gal concentration increased from 20 to 80 mg/mL (Figure 7A). Cellular senescence markers were subsequently examined. The protein expression levels of GLB1, p53, and p21 increased progressively with increasing doses of D‐gal, indicating that d‐gal induced the upregulation of senescence‐associated proteins (Figure 7B,C). The proportion of SA‐β‐gal–positive cells also increased with higher d‐gal concentrations, suggesting an exacerbation of cellular senescence (Figure 7D,E). Moreover, fluorescence intensity detected using the Senescence‐Tracker probe was significantly enhanced as d‐gal concentrations increased, further confirming the concentration‐dependent induction of cellular senescence by d‐gal (Figure 7F,G). Overall, d‐gal inhibited cell viability and induced cellular senescence. We therefore selected a 40 mg/mL concentration of d‐gal for follow‐up experiments, as it elicited a strong senescence phenotype with acceptable cell survival.

#### 3.5.2 Pathological index detection in MN9D cells

**FIGURE 8:**
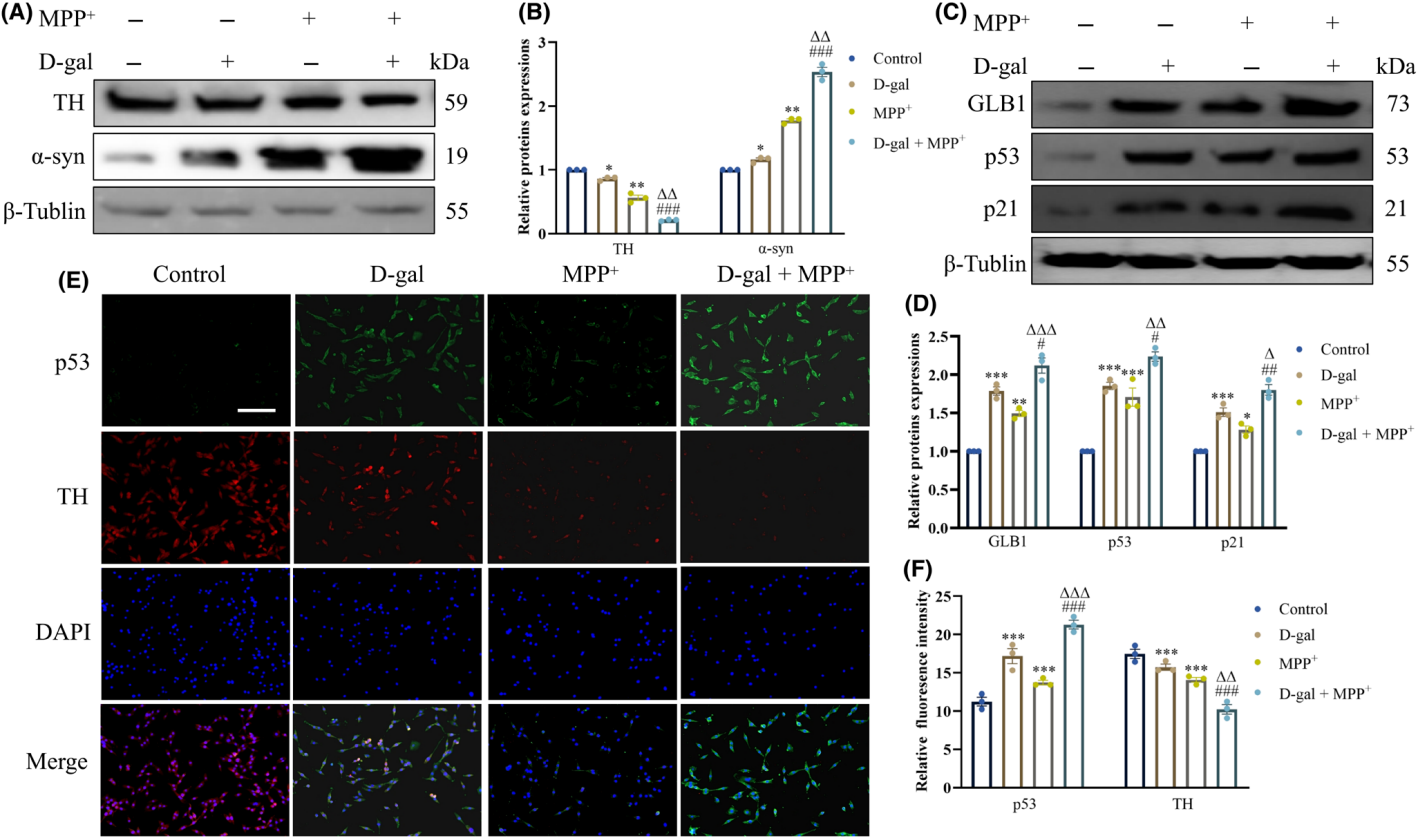
Pathology index detection of MN9D cell. (A) Western blot of Parkinson's disease (PD)‐related proteins tyrosine hydroxylase (TH) and α‐synuclein (α‐syn) (β‐tubulin as internal control). (B) Relative protein quantification of panel A. (C) Western blot of senescence‐related proteins GLB1, p53, p21 (β‐tubulin as internal control). (D) Relative protein quantification of panel C. (E) Immunofluorescence co‐localization of p53 and TH in MN9D cells, DAPI for nuclear staining (scale bar = 50 μm). (F) Quantification of mean fluorescence intensity of TH and p53. *n* = 3 biological replicates per group; data are presented as mean ± standard deviation (SD). One‐way analysis of variance (ANOVA) with Tukey's honestly significant difference (HSD) post‐hoc test and Bonferroni's correction. *p < 0.05, **p < 0.01, ***p < 0.001 versus control; ^#^ *p* < 0.05, ^##^ *p* < 0.01, ^###^ *p* < 0.001 versus d‐gal; ^∆^ *p* < 0.05, ^∆∆^ *p* < 0.01, ^∆∆∆^ *p* < 0.001 versus MPP^+^.

The d‐gal, MPP^+^, and d‐gal + MPP^+^ groups showed decreased TH expression and increased α‐syn, GLB1, p53, and p21 expressions compared to the control group, with the most significant alterations in the d‐gal + MPP^+^ group (Figure 8A–D). Immunofluorescence analysis revealed co‐localization of p53 with TH and p21 with α‐syn. Consistent with protein expression data, the average fluorescence intensity of TH was reduced, whereas the intensities of p53, p21, and α‐syn were increased (Figures 8E,F and S4A,B). SA‐β‐Gal staining and senescence‐associated fluorescent probe detection further demonstrated that, compared to the control group, senescence‐associated fluorescence intensity was significantly increased in the d‐gal, MPP^+^, and d‐gal + MPP^+^ groups (Figure S4C,D), confirming accelerated cellular senescence under these treatment conditions. In addition, cellular ROS levels were measured, and the average ROS fluorescence intensity in these groups was higher than that in the control group (Figure S4E,F), indicating increased oxidative stress in cells due to increased ROS production.

#### 3.5.3 Hub gene analysis

**FIGURE 9:**
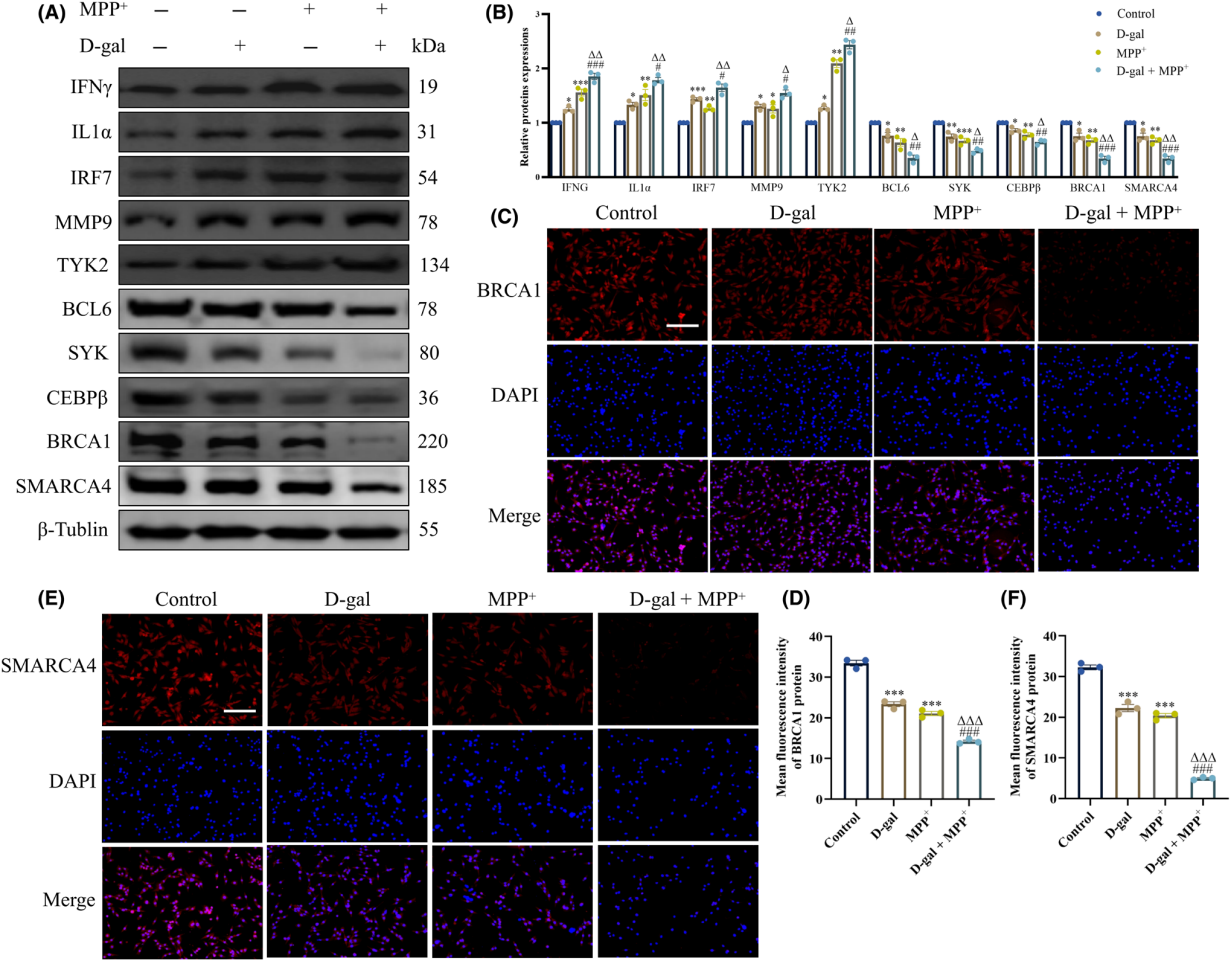
Hub gene analysis. (A) Western blot of 10 hub proteins (IFNγ, IL1α, IRF7, MMP9, TYK2, BCL6, SYK, CEBPβ, BRCA1, SMARCA4), with β‐tubulin as internal control. (B) Relative protein quantification of the 10 hub genes. (C) Immunofluorescence staining of BRCA1 (red, DAPI for nuclear staining, scale bar = 50 μm). (D) Quantification of BRCA1 mean fluorescence intensity. (E) Immunofluorescence staining of SMARCA4 (red, DAPI for nuclear staining, scale bar = 50 μm). (F) Quantification of SMARCA4 mean fluorescence intensity. *n* = 3 biological replicates per group; data are presented as mean ± standard deviation (SD). One‐way analysis of variance (ANOVA) with Tukey's honestly significant difference (HSD) post‐hoc test and Bonferroni's correction. *p < 0.05, **p < 0.01, ***p < 0.001 versus control; ^#^ *p* < 0.05, ^##^ *p* < 0.01, ^###^ *p* < 0.001 versus d‐gal; ^∆^ *p* < 0.05, ^∆∆^ *p* < 0.01, ^∆∆∆^ *p* < 0.001 versus MPP^+^.

Compared to the control group, the expression levels of IFNγ, IL1α, IRF7, MMP9, and TYK2 were upregulated in the d‐gal or MPP^+^ groups, whereas BCL6, SYK, CEBPβ, BRCA1, and SMARCA4 were downregulated (Figure 9A,B). These expression trends were more pronounced in the d‐gal + MPP^+^ group compared to the d‐gal or MPP^+^ groups. Immunofluorescence analysis further demonstrated that the average fluorescence intensities of BRCA1 and SMARCA4 in MN9D cells were reduced in the d‐gal or MPP^+^ groups compared to the control group, with an even greater reduction observed in the d‐gal + MPP^+^ group (Figure 9C–F).

#### 3.5.4 IRF7 as a candidate biomarker for aging, combined with PD

**FIGURE 10:**
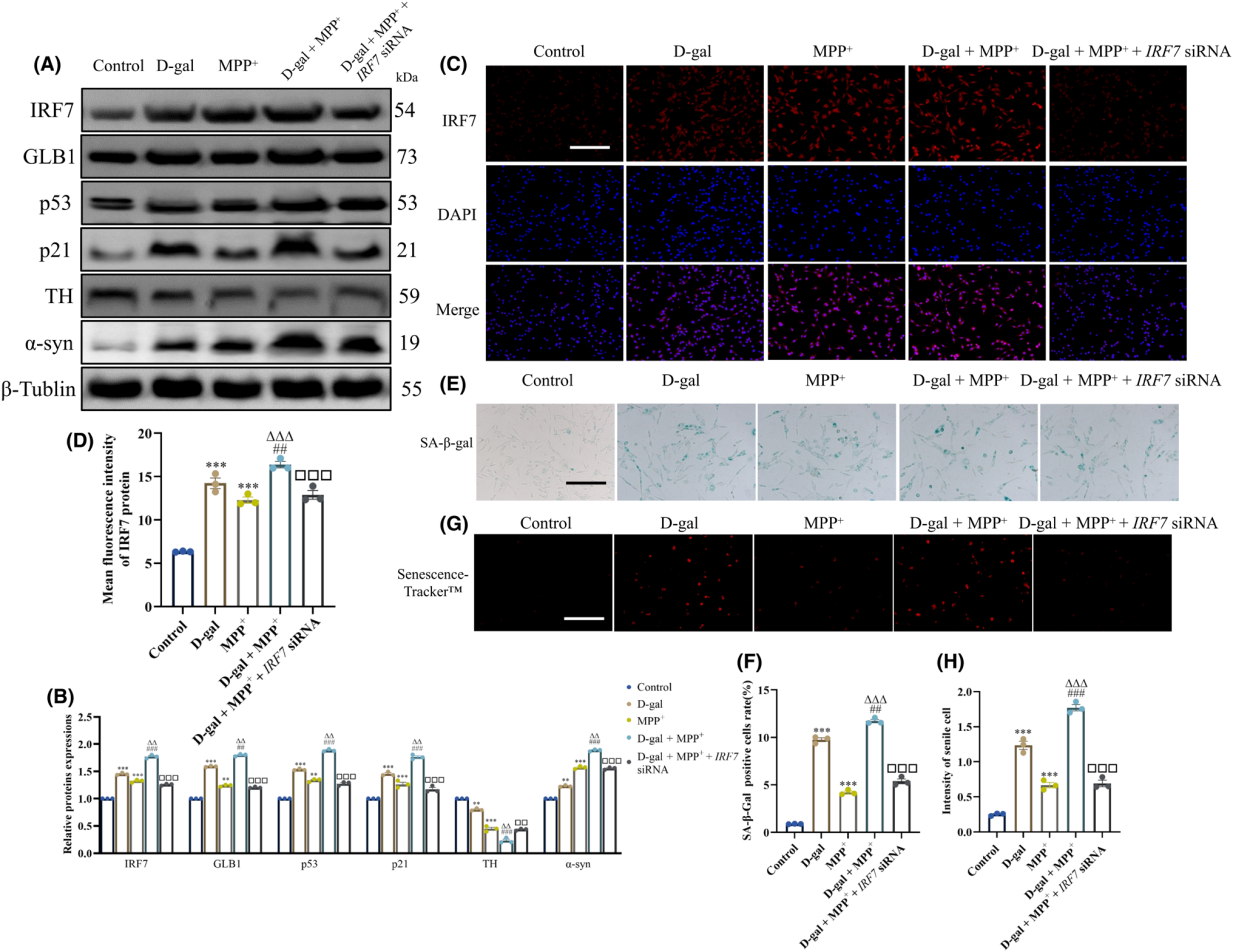
IRF7 as a biomarker for diagnosing aging combined with Parkinson's disease (PD). (A) Western blot of IRF7, senescence‐related proteins (GLB1, P53, P21), and PD‐related proteins (TH, α‐syn), with β‐tubulin as internal control (B) Relative protein quantification of panel A. (C) Immunofluorescence staining of IRF7 (red, DAPI for nuclear staining, scale bar = 50 μm). (D) Quantification of IRF7 mean fluorescence intensity. (E) SA‐β‐gal staining of senescent MN9D cells (scale bar = 50 μm). (F) Quantification of SA‐β‐gal–positive cell percentage. (G) Senescence‐Tracker fluorescent probe detection of senescent cells (scale bar = 50 μm). (H) Quantification of relative fluorescence intensity. *n* = 3 biological replicates per group; data are presented as mean ± standard deviation (SD). One‐way analysis of variance (ANOVA) with Tukey's honestly significant difference (HSD) post‐hoc test and Bonferroni's correction. **p < 0.01, ***p < 0.001 versus control; ^##^ *p* < 0.01, ^###^ *p* < 0.001 versus d‐gal; ^∆∆^ *p* < 0.01, ^∆∆∆^ *p* < 0.001 versus MPP^+^; ^□□^ *p* < 0.01, ^□□□^ *p* < 0.001 versus d‐gal + MPP^+^.

Compared to the control group, the expression levels of IRF7, GLB1, p53, p21, and α‐syn were increased in the d‐gal and MPP^+^ groups, whereas TH expression was reduced. These changes were more pronounced in the d‐gal + MPP^+^ group, which exhibited significantly higher levels of the aforementioned proteins and a further decrease in TH expression. In contrast, relative to the d‐gal + MPP^+^ group, the d‐gal + MPP^+^ + *IRF7* siRNA group showed reduced expression of IRF7, senescence‐associated proteins, and α‐syn, accompanied by increased TH expression (Figure 10A,B). These results suggest that silencing IRF7 can regulate senescence and PD‐related biomarkers. Immunofluorescence analysis demonstrated that IRF7 fluorescence intensity was notably higher in the d‐gal, MPP^+^, and d‐gal + MPP^+^ groups compared to the control group, indicating increased IRF7 protein levels in aging‐ and PD‐associated cellular models. Notably, treatment with *IRF7* siRNA in the d‐gal + MPP^+^ group markedly reduced IRF7 fluorescence intensity (Figure 10C,D). The percentage of SA‐β‐gal–positive cells was markedly elevated in the d‐gal, MPP^+^, and d‐gal + MPP^+^ groups relative to the control group. In contrast, the d‐gal + MPP^+^ + *IRF7* siRNA group exhibited a lower positive cell rate than the d‐gal + MPP^+^ group. As a classical marker of cellular senescence, SA‐β‐gal activity changed closely with IRF7 expression, suggesting that IRF7 participates in senescence‐related processes underlying PD pathogenesis (Figure 10E,F). Similarly, senescence‐associated fluorescence intensity was elevated in the d‐gal, MPP^+^, and d‐gal + MPP^+^ groups compared to the control group but was significantly reduced following *IRF7* knockdown. These results further demonstrate that PD‐related treatments enhance cellular senescence and that IRF7 expression closely correlated with cellular senescence (Figure 10G,H).

#### 3.5.5 Prediction of therapeutic drugs and molecular docking

**FIGURE 11:**
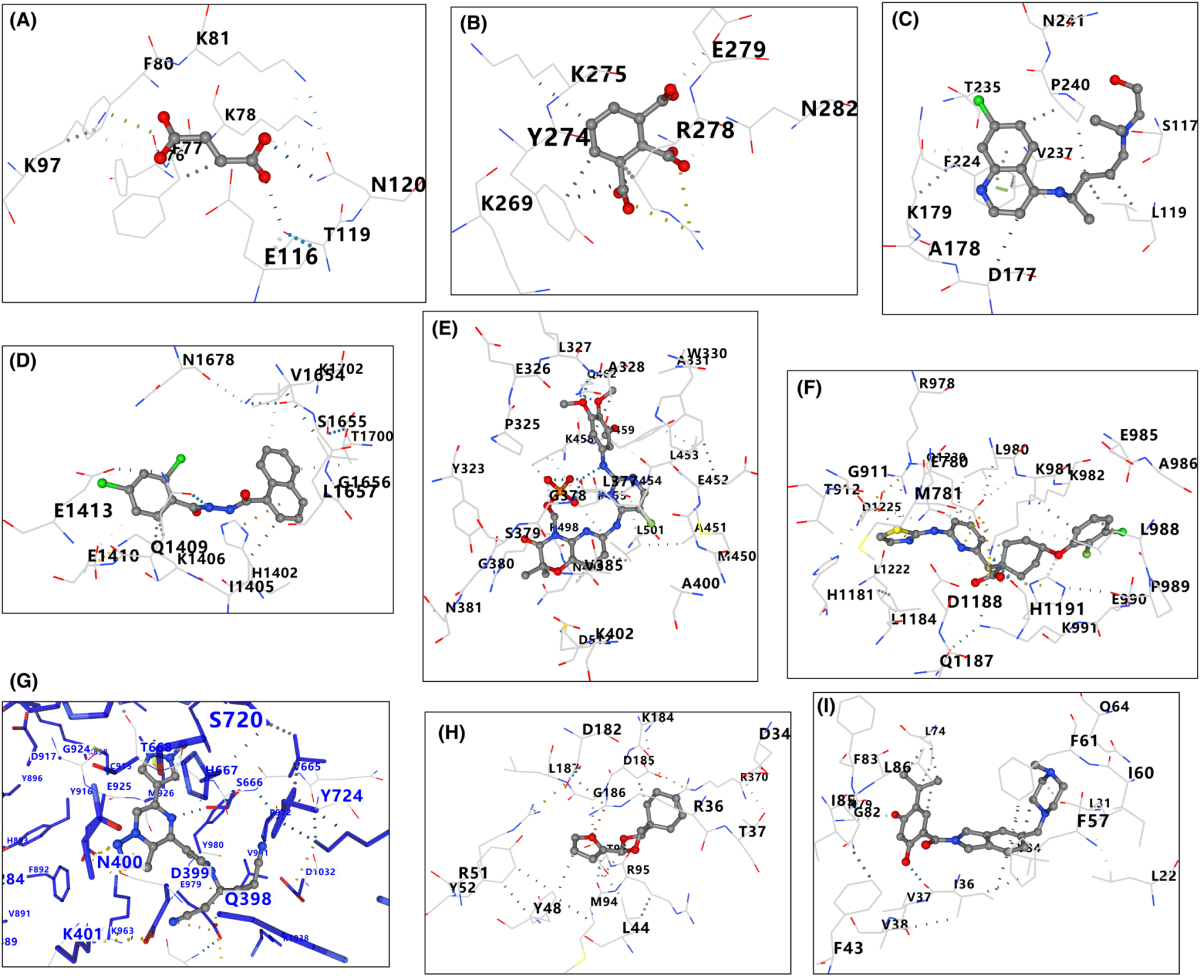
Screening the binding mode between drugs and targets through molecular docking. (A) Fumaric acid molecular docking with IFNγ. (B) 1,2,3‐benzenetricarboxylic acid molecular docking with CEBPβ. (C) Hydroxychloroquine molecular docking with IL1α. (D) CHEMBL1709259 molecular docking with BRCA1. (E) Fostamatinib molecular docking with SYK. (F) MK‐5108molecular docking with SMARCA4. (G) Ropsacitinib molecular docking with TYK2. (H) Ulinastatin molecular docking with MMP9. (I) Onalespib molecular docking with BCL6.

We predicted therapeutic drugs targeting hub proteins via the DGIdb database, as summarized in Table S3. Molecular docking was employed to determine the binding affinity of the potential compounds toward their respective protein targets. The results showed that all drug candidates formed stable interactions with their targets via hydrogen bonding and robust electrostatic interactions (Figures 11A‐I and S5). Specifically, the binding energies for IFNγ, CEBPβ, IL1α, BRCA1, SYK, SMARCA4, TYK2, MMP9, and BCL6 were −4.1, −5.1, −5.5, −7.5, −9.8, −9.0, −9.4, −6.6, and −8.5 kcal/mol, respectively, indicating favorable and stable binding interactions. The principal amino acid residues implicated in these interactions are enumerated in Table 1.

**TABLE 1:** The binding energy and amino acid sites of drug molecular docking with protein.

Protein	Drug	Binding energy (kcal/mol)	Amino acid site
IFNγ (AF‐P01579‐F1)	Fumaric acid	−4.1	Chain A: VAL73 SER74 PHE75 TYR76 PHE77 LYS78 PHE80 LYS81 PHE83 LYS84 GLN90 VAL93 GLU94 LYS97 PHE115 GLU116 LYS117 THR119 ASN120
CEBPβ (AF‐P17676‐F1)	1,2,3‐Benzenetricarboxylic acid	−5.1	Chain A: PHE75 TYR78 LYS269 TYR274 LYS275 ARG278 GLU279 ASN281 ASN282 ILE283 VAL285 ARG286 ARG289 ASP290 LYS293
IL1α (AF‐P01583‐F1)	Hydroxychloroquine	−5.5	Chain A: SER117 LEU119 VAL122 TYR124 LYS175 ASP176 ASP177 ALA178 LYS179 PHE224 GLU226 HIS228 THR235 VAL237 PRO240 ASN241 PHE243
BRCA1 (AF‐P38398‐F1)	CHEMBL1709259	−7.5	Chain A: ASP1398 GLN1401 HIS1402 ILE1405 LYS1406 GLN1409 GLU1410 GLU1413 VAL1654 SER1655 GLY1656 LEU1657 THR1658 GLU1661 ASN1678 LEU1679 LYS1690 THR1691 ASP1692 VAL1696 CYS1697 GLU1698 ARG1699 THR1700 LEU1701 LYS1702
SYK (AF‐P43405‐F1)	Fostamatinib	−9.8	Chain A: PHE320 ASN321 PRO322 TYR323 GLU324 PRO325 GLU326 LEU327 ALA328 PRO329 TRP330 ALA331 ALA332 LEU377 GLY378 SER379 GLY380 ASN381 PHE382 GLY383 VAL385 ALA400 LYS402 MET450 ALA451 GLU452 LEU453 GLY454 PRO455 ASN457 LYS458 TYR459 GLN461 GLN462 ASP494 ALA497 ARG498 ASN499 LEU501 SER511 ASP512 GLY532 LYS533 TRP534 PRO535 VAL536 LYS537 TRP538 GLU564 LYS571
SMARCA4 (AF‐P51532‐F1)	MK‐5108	−9.0	Chain A: GLU780 MET781 GLY911 THR912 ARG978 LEU980 LYS981 LYS982 GLU985 ALA986 GLN987 LEU988 PRO989 GLU990 LYS991 VAL992 GLU993 HIS1181 LEU1184 GLN1187 ASP1188 HIS1191 LEU1222 ASP1225 GLN1226 GLN1230
TYK2 (AF‐P29597‐F1)	Ropsacitinib	−9.4	Chain A: ARG281 LEU282 LEU283 ALA284 ALA286 GLU287 GLY288 GLU289 TYR376 ASP382 ARG397 GLN398 ASP399 ASN400 LYS401 GLU404 GLN664 VAL665 SER666 HIS667 THR668 LEU670 ALA671 PHE672 TYR724 ASN727 LYS728 PHE754 LYS756 ASP888 PRO889 VAL891 PHE892 HIS893 TYR896 TYR916 ASP917 PRO918 THR919 ASP921 THR923 GLY924 GLU925 MET926 LYS961 LYS963 GLU979 TYR980 PRO982 ASP1032
MMP9 (AF‐P14780‐F1)	Ulinastatin	−6.6	Chain A: GLY33 ASP34 LEU35 ARG36 THR37 LEU44 TYR48 ARG51 TYR52 MET94 ARG95 THR96 ALA173 PHE181 ASP182 GLY183 LYS184 ASP185 GLY186 LEU187 ASP207 GLY339 GLY340 ARG366 GLY367 ASP368 ARG370 TRP385 PHE387
BCL6 (AF‐P41182‐F1)	Onalespib	−8.5	Chain A: PHE11 LEU19 LEU22 LEU31 THR32 ASP33 VAL34 VAL35 ILE36 VAL37 VAL38 PHE43 LEU50 PHE57 ILE60 PHE61 GLN64 LEU65 ASN68 ILE72 LEU74 ASP75 PRO76 ILE78 ASN79 PRO80 GLU81 GLY82 PHE83 CYS84 ILE85 LEU86 PHE89

**FIGURE 12:**
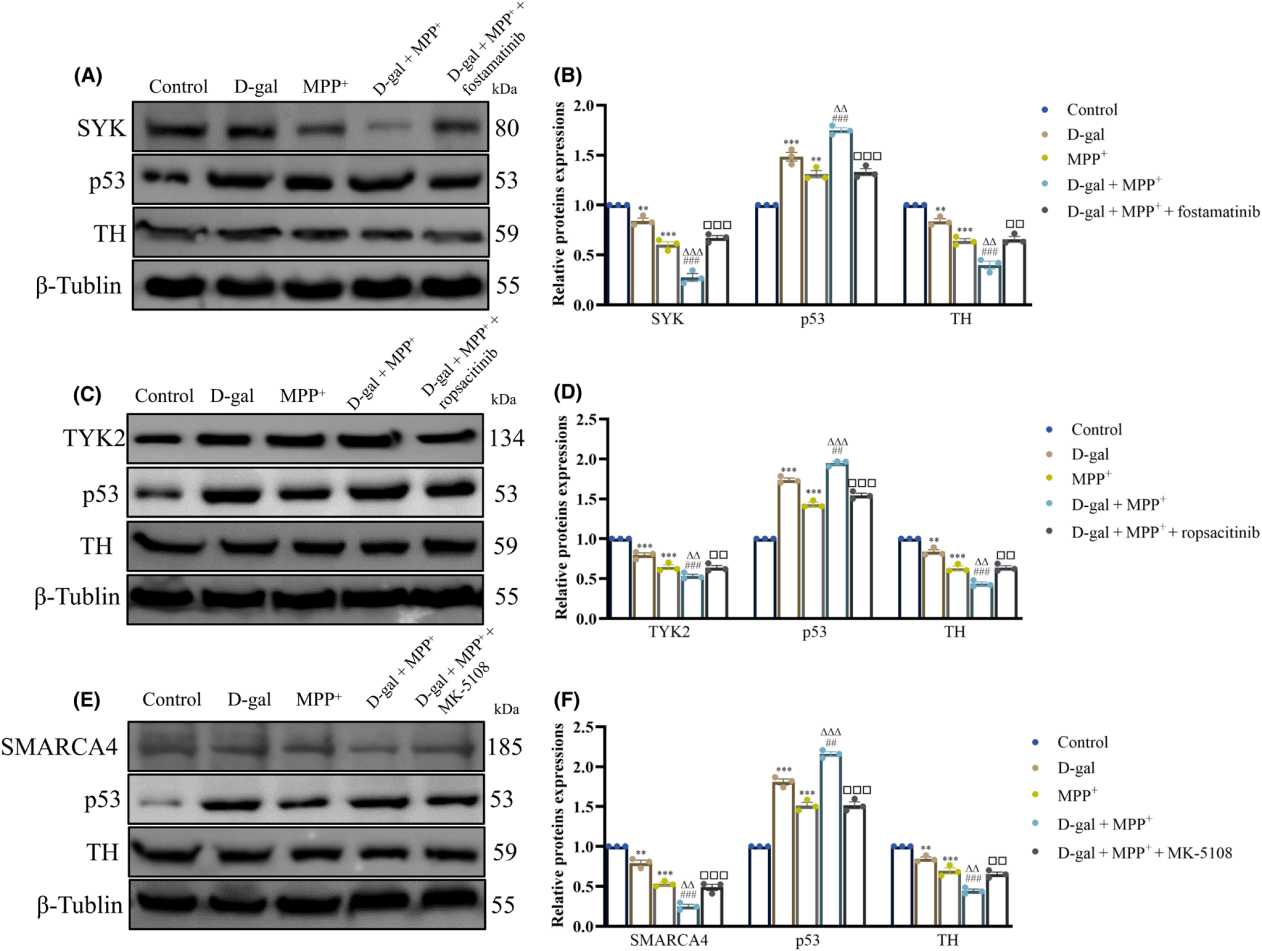
Analysis of the therapeutic effect of cellular models of aging and PD, including fostamatinib, ropsacitinib, and mk‐5108. (A) Western blot of target protein SYK, senescence marker p53, and PD marker TH in fostamatinib‐treated cells (β‐tubulin as internal control). (B) Relative protein quantification of panel A. (C) Western blot of target protein TYK2, p53, and TH in ropsacitinib‐treated cells (β‐tubulin as internal control). (D) Relative protein quantification of panel C. (E) Western blot of target protein SMARCA4, p53, and TH in MK‐5108–treated cells (β‐tubulin as internal control). (F) Relative protein quantification of panel E. *n* = 3 biological replicates per group; data are presented as mean ± standard deviation (SD). One‐way analysis of variance (ANOVA) with Tukey's honestly significant difference (HSD) post‐hoc test and Bonferroni's correction. **p < 0.01, ***p < 0.001 versus control; ^##^ *p* < 0.01, ^###^ *p* < 0.001 versus d‐gal; ^∆∆^ *p* < 0.01, ^∆∆∆^ *p* < 0.001 versus MPP^+^; ^□□^ *p* < 0.01, ^□□□^ *p* < 0.001 versus d‐gal + MPP^+^.

Compared to the control group, SYK protein expression was decreased in the d‐gal and MPP^+^ groups and significantly reduced in the d‐gal + MPP^+^ group. Notably, SYK expression was restored following fostamatinib treatment in the d‐gal + MPP^+^ model (Figure 12A,B). Compared to the control group, TYK2 protein expression was elevated in the d‐gal and MPP^+^ groups and further increased in the d‐gal + MPP^+^ group, whereas ropsacitinib treatment significantly reduced TYK2 expression (Figure 12C,D). Similarly, SMARCA4 protein expression was decreased in the d‐gal and MPP^+^ groups, with the most pronounced reduction in the d‐gal + MPP^+^ group. MK‐5108 treatment partially restored SMARCA4 expression (Figure 12E,F). In all aging and PD model groups, p53 expression was increased and TH expression was decreased compared to the control group. Drug treatment reversed these trends, yielding lower p53 levels and higher TH expression (Figure 12). In summary, fostamatinib, ropsacitinib, and MK‐5108 exerted specific regulatory effects on senescence‐related and PD‐related proteins in cellular models, highlighting their potential therapeutic value.

## 4 DISCUSSION

PD is a prevalent neurodegenerative disorder that severely impairs patients' quality of life. With accelerating global population aging, the incidence of PD continues to increase. The present study focused on the association between SRGs and PD, with the aim of clarifying the molecular mechanisms underlying aging‐related processes in PD and exploring preliminary directions for PD diagnosis and intervention. Integrated analysis of multiple transcriptomic datasets identified 2925 DEGs and 31 DESRGs. KEGG enrichment analysis revealed that these DESRGs were significantly enriched in multiple signaling pathways, including the JAK–STAT and IL‐17, among others. These results suggest that SRGs do not function independently during PD pathogenesis but interact within complex regulatory signaling networks.

The JAK–STAT signaling pathway plays critical roles in diverse physiological processes, including cell proliferation, differentiation, and immune regulation. Under physiological conditions, this pathway is tightly regulated to maintain normal neuronal function. However, aberrant activation of the JAK–STAT pathway has been reported in PD.^25^ Such dysregulation disrupts fundamental neuronal physiology, particularly impairing neurotransmitter synthesis and synaptic transmission.^26^ Neurotransmitter production relies on a series of tightly controlled biochemical cascades, and abnormal JAK–STAT signaling can disturb this homeostatic balance. For instance, sustained JAK–STAT activation may suppress TH expression by modulating the activity or transcription of key enzymes, consequently reducing dopamine biosynthesis.^25^ Furthermore, dysregulated JAK–STAT signaling interferes with synaptic plasticity and nerve impulse transmission, diminishing signaling fidelity to downstream neurons and ultimately triggering neuronal dysfunction.^27^ Cumulatively, persistent neuronal injury drives the progression of PD, gradually leading to the deterioration of motor and cognitive functions.

The AGE–RAGE signaling axis is critically involved in oxidative stress and inflammatory responses during aging. With advancing age, AGEs progressively accumulate in tissues and bind to their cell surface receptor RAGE, thereby activating the AGE–RAGE signaling cascade.^28^ In PD, this pathway is notably hyperactivated. AGE–RAGE signaling stimulates excessive intracellular ROS generation, leading to exacerbated oxidative stress. Overproduced ROS directly damage neuronal membranes, proteins, and DNA, thereby impairing the structural and functional integrity of dopaminergic neurons. Furthermore, AGE–RAGE activation promotes chronic neuroinflammation by inducing the expression of pro‐inflammatory cytokines, including TNF‐α and IL‐6.^29^, ^30^ This persistent inflammatory milieu further injures neurons, disturbs neurotransmitter metabolism and synaptic transmission, aggravates neuronal loss and dysfunction, and ultimately accelerates the pathological progression of PD.

The HIF‐1 signaling pathway serves as a key adaptive mechanism that enables cellular responses to hypoxic stress. Under physiological conditions, the expression and activity of HIF‐1 are tightly controlled to maintain oxygen homeostasis. However, during PD pathogenesis, abnormal neuronal metabolism, local ischemia, and other stressors trigger aberrant activation of the HIF‐1 pathway.^31^ HIF‐1α, the core regulatory subunit of this pathway, is stabilized under hypoxia or cellular stress, translocates into the nucleus, and binds to hypoxia‐responsive elements to regulate the transcription of downstream target genes. In PD, dysregulated HIF‐1α signaling alters the expression of genes governing metabolism, angiogenesis, and cell survival. For example, upregulation of vascular endothelial growth factor may partially alleviate local ischemia; however, it can also trigger aberrant angiogenesis, impair blood–brain barrier integrity, and promote the infiltration of neurotoxic substances into the brain, thereby exacerbating neuronal damage.^32^, ^33^ Furthermore, HIF‐1α dysregulation may disrupt neuronal energy metabolism by altering metabolic gene expression, leading to insufficient energy supply and accelerated PD progression.

The IL‐17 signaling pathway exerts a key pro‐inflammatory function in immune regulation. During PD progression, this pathway is activated, accompanied by upregulated IL‐17α expression. IL‐17α acts on multiple cell types, including neurons and glial cells, to induce the production of pro‐inflammatory cytokines and chemokines such as IL‐6, IL‐1β, and CXCL8, thereby triggering prominent inflammatory responses.^34^ In neurons, IL‐17α‐mediated inflammation impairs neurotransmitter synthesis and release, thereby disrupting normal neuronal function. Meanwhile, this inflammatory cascade activates immune cells, which further attack neurons and promote neuronal damage and apoptosis.^35^, ^36^ Sustained activation of the IL‐17 pathway forms a self‐reinforcing vicious cycle that continuously amplifies neuroinflammation and exacerbates neurodegenerative alterations in PD, ultimately leading to progressive disease deterioration. As a central intracellular regulator, the NF‐κB signaling pathway orchestrates inflammatory responses. Under physiological conditions, NF‐κB signaling remains largely inactive to maintain intracellular homeostasis. In PD, however, oxidative stress and inflammatory stimuli drive aberrant activation of the NF‐κB pathway. Upon activation, NF‐κB translocates from the cytoplasm to the nucleus, where it binds to promoter regions and modulates the transcription of pro‐inflammatory genes. These genes encode inflammatory cytokines (e.g., TNF‐α, IL‐1β, and IL‐6), chemokines, and adhesion molecules.^37^ Massive release of these inflammatory mediators triggers an amplification cascade, which disrupts the neuronal microenvironment and triggers neuroinflammation. This disturbed microenvironment not only directly injures neurons but also impairs interneuronal signal transmission. Imbalanced neurotransmission further contributes to neuronal dysfunction and eventual cell death.^38^ Moreover, persistent NF‐κB activation compromises cellular antiapoptotic defenses, rendering neurons more susceptible to damage, thereby contributing critically to PD onset and progression.^39^

The TNF signaling pathway, mainly mediated by TNF‐α and its receptors TNFR1 and TNFR2, also contributes critically to PD pathogenesis. During PD progression, TNF‐α expression is markedly upregulated, leading to the activation of the TNF signaling cascade. This activation triggers multiple intracellular events, among which is further activation of the NF‐κB pathway, which in turn amplifies inflammatory responses and exacerbates neuronal damage.^40^ Additionally, TNF signaling promotes neuronal apoptosis. After binding to TNFR1, TNF‐α recruits adaptor proteins to form the death‐inducing signaling complex (DISC), activates caspase family proteins, and initiates a caspase cascade that ultimately drives apoptosis.^41^, ^42^ Furthermore, TNF signaling disrupts intracellular redox balance by stimulating ROS production, enhances oxidative stress, and further compromises neuronal structure and function, thereby accelerating PD progression.^43^ Collectively, these interconnected signaling pathways coordinately regulate neuronal function and survival. A thorough understanding of their cross talk and mechanisms is essential for elucidating PD etiology and developing targeted therapeutic strategies.

Among the hub genes identified through PPI analysis, *IRF7* exhibited consistent abnormal expression in both PD cells and animal models. In MN9D cells treated with d‐gal + MPP^+^ and in d‐gal + MPTP‐induced mouse models, *IRF7* expression was significantly elevated, accompanied by accelerated cellular senescence and worsening of PD‐related phenotypes. siRNA‐mediated knockdown of *IRF7* partially restored TH expression, reduced α‐syn aggregation, and alleviated levels of senescence markers in cellular models. These findings suggest that *IRF7* may serve as a candidate molecular marker mediating cellular senescence and dopaminergic neuron degeneration. However, this study provides only correlational and partial functional evidence; further experiments involving overexpression and detailed mechanistic analysis are required to confirm whether *IRF7* is a key driver of aging‐related PD‐like pathology.

Behavioral and pathological results show that, compared to single treatments, combined d‐gal + MPTP treatment induces more severe motor deficits, dopaminergic neuron loss, α‐syn aggregation, and cellular senescence. This model reproduces the core features of stress‐induced premature aging and PD‐like neurodegeneration. It should be noted that d‐gal treatment simulates a stress‐induced senescence‐like state rather than true physiological aging; therefore, conclusions regarding “accelerated aging and PD progression” must be interpreted with caution in light of this limitation. This study supports the view that stress‐related premature aging can exacerbate the pathological progression of PD, but it does not fully mimic the natural aging process.

Through virtual drug screening and molecular docking, this study further identified candidate small‐molecule compounds targeting hub genes, including fostamatinib, ropsacitinib, and MK‐5108. In vitro experiments demonstrated that these compounds can partially restore target protein expression in MN9D cells and improve aging‐ and PD‐related phenotypes. However, these pharmacological findings remain at the preliminary exploratory and hypothesis‐generating stage. This study has not yet confirmed whether the observed effects are achieved through direct binding to targets or specific targeting mechanisms; further clarification of their therapeutic potential requires the use of gene knockout/overexpression systems, in vivo efficacy validation, and studies on target‐specific mechanisms.

Recent studies have explored the pathogenesis of PD from multiple perspectives, complementing and supporting the findings of this research. Research indicates that alterations in gut microbiota are closely linked to the initiation and progression of PD, and that impaired gut barrier function and immune activation may influence neuronal cells in the brain through the neuro–immune–endocrine axis.^44^, ^45^ These observations are consistent with the activation of inflammation‐related signaling pathways, such as the NF‐κB pathway, identified in this study, highlighting the central role of inflammatory responses in PD pathogenesis and suggesting potential interactions across multiple biological systems. Genome‐wide association studies have uncovered multiple genetic loci linked to PD at the genetic level.^46^, ^47^ Although these loci differ from the hub genes identified in this research, the collective evidence indicates that genetic diversity plays a significant role in PD development. This suggests that different genetic research approaches can uncover distinct aspects of the genetic basis of PD, together offering a more comprehensive understanding of its pathogenesis. Furthermore, recent advances have highlighted the role of mitochondrial dysfunction in PD. Mitochondria, the primary energy‐producing organelles in cells, are capable of disrupting energy metabolism and elevating oxidative stress.^48^, ^49^, ^50^ This finding is consistent with the cellular senescence and oxidative stress observed in the present study, further supporting the critical role of oxidative stress in PD pathogenesis and suggesting that mitochondrial dysfunction may represent a key mechanistic link between aging and PD.

With respect to inflammation and immune regulation, this research identified the activation of multiple inflammation‐related signaling pathways, indicating that inflammatory responses play a pivotal role in PD initiation. Future studies may further investigate the interactions between inflammatory cytokines and SRGs to determine whether modulation of inflammatory responses could be an effective strategy for intervening in PD progression. For example, targeted therapies against specific inflammatory mediators could be developed to suppress excessive activation of inflammatory signaling pathways, thereby reducing neuronal damage. In terms of gene regulatory networks, although this study identified several key genes and signaling pathways, the upstream and downstream regulatory relationships among these genes remain to be fully elucidated. Gene‐editing techniques, such as CRISPR‐Cas9, could be employed to functionally validate hub genes and investigate their regulatory effects on downstream gene expression and their specific roles in PD pathogenesis. Such efforts would contribute to the construction of a more comprehensive gene regulatory network model for PD. From a multiomics perspective, the integration of genomes, transcriptomics, proteomics, and metabolomics may yield a more comprehensive knowledge of the molecular changes driving PD pathogenesis. For example, metabolomic analyses can identify metabolic changes in PD patients, revealing potential associations between metabolic abnormalities and aging‐related genes and offering additional clues for identifying novel therapeutic targets.

This study has several limitations: First, the diagnostic value of the hub gene has only been evaluated in a limited public dataset and has not been independently validated in clinical settings; its application as an early diagnostic biomarker remains in the preliminary stage. Second, the data on the *IRF7* mechanism are incomplete, lacking overexpression or rescue experiments to establish causality. Third, the d‐gal + MPTP model reflects stress‐induced aging rather than physiological aging; fourth, the candidate drugs lack clear validation of target‐dependent mechanisms; fifth, all experiments were conducted in cellular and mouse systems, and translation to human PD requires further validation in clinical samples.

In summary, this study preliminarily confirms the abnormal regulation of aging‐related genes and associated inflammatory signaling pathways in PD‐like models; *IRF7* is a candidate molecule mediating aging‐related dopaminergic neuron degeneration, and various small molecules can serve as exploratory tool compounds for subsequent validation. These findings provide resources for elucidating the association between cellular aging and PD and offer candidate molecular targets for further mechanistic studies and translational research.

## 5 CONCLUSION

**FIGURE 13:**
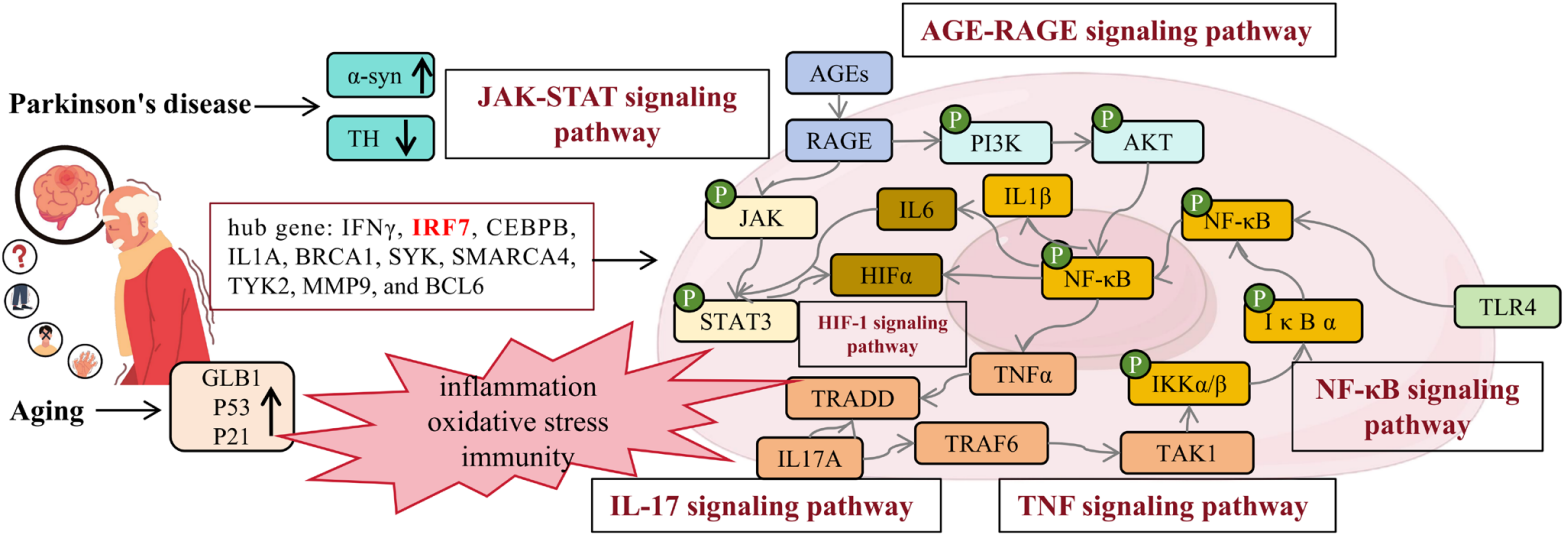
Molecular mechanisms and signaling pathways in Parkinson's disease (PD) and aging. PD increases α‐synuclein (α‐syn) and decreases tyrosine hydroxylase (TH), whereas aging boosts GLB1, p53, and p21. Hub genes regulate these pathways. α‐syn and TH changes may activate JAK–STAT. AGEs bind to RAGE, activating PI3K‐AKT, influencing inflammation. HIFα upregulation engages the HIF‐1 pathway in hypoxic responses. IL17 promotes inflammation through signaling. These pathways interact, affecting inflammation, oxidative stress, immune responses, neuronal function, and PD progression, linking aging to PD pathology. TNF‐α activates TAK1, initiating signal transduction, apoptosis, inflammation, and interacting with other pathways, reflecting the tight connection between aging and PD processes.

At the genomic level, a large number of PD and aging‐related DEGs were identified. Ten hub genes, especially *IRF7* and *IFNγ*, were identified. These genes are expected to become candidate targets for PD intervention and preliminary auxiliary diagnostic biomarkers, but their diagnostic performance and causal effects still need to be further validated in independent clinical cohorts and comprehensive functional experiments. From the perspective of signaling pathways, multiple pathways—such as JAK–STAT, AGE–RAGE, HIF‐1, IL‐17, NF‐κB, and TNF—are activated during PD progression and exhibit extensive cross talk (Figure 13). These pathways disrupt neuronal homeostasis and contribute to PD development through mechanisms involving inflammation, oxidative stress, and immune responses. Moreover, age‐related alterations in the expression of key regulatory factors interact with PD pathological processes, underscoring the pivotal role of aging in PD pathogenesis. Notably, the d‐gal model reflects stress‐induced senescence rather than physiological natural aging, and the role of physiological aging in PD pathogenesis remains to be verified.

## AUTHOR CONTRIBUTIONS

**Haojie Wu:** Data curation; formal analysis; methodology; writing – review and editing; writing – original draft. **Jianshe Wei:** Conceptualization; funding acquisition; writing – review and editing. **Xiangshu Cheng:** Methodology. **Tingting Liu:** Conceptualization; data curation; formal analysis; funding acquisition; writing – original draft.

## FUNDING INFORMATION

This work was supported partly by National Natural Science Foundation of China (32161143021, 81271410), Henan Provincial Science and Technology Key Projects Program (252102311160, 252301420027), Henan Provincial Medical Science and Technology Key Projects Program (SBGJ202402075), and Henan University Graduate “Talent Program” of Henan Province (SYLYC2023092).

## CONFLICT OF INTEREST STATEMENT

The authors declare that there is no conflict of interest regarding the publication of this paper.

## ETHICS STATEMENT

The experiments involving mice conducted in this study were approved by the Institutional Animal Care and Use Committee at Henan University and were carried out in accordance with the committee's guidelines (approval no.: HUSOM2021‐161). Furthermore, this study strictly adhered to the ARRIVE 2.0 guidelines.

## Supporting information


**Figure S1.** Gene chip data information. (A) The gene chip expression profiles of 43 brain tissue samples, including 26 Parkinson's disease (PD) patients and 17 healthy patients. (B) Principal component analysis (PCA) analysis. (C) The differential gene volcano map. Red represents upregulated genes, and blue represents downregulated genes.
**Figure S2.** (A) Body weight curve of mice in each group over 8 weeks, *n* = 10 per group. (B) Immunofluorescence staining of tyrosine hydroxylase (TH) in mouse substantia nigra (SN) (red, DAPI for nuclear staining, scale bar = 100 μm). (C) Quantification of TH mean fluorescence intensity, *n* = 3 per group. Data are presented as mean ± standard deviation (SD). One‐way analysis of variance (ANOVA) with Tukey's honestly significant difference (HSD) post‐hoc test and Bonferroni's correction. *p < 0.05, **p < 0.01, ***p < 0.001 versus saline; ^#^ *p* < 0.05, ^##^ *p* < 0.01, ^###^ *p* < 0.001 versus d‐gal; ^∆^ *p* < 0.05 versus 1‐methyl‐4‐phenyl‐1,2,3,6‐tetrahydropyridine (MPTP).
**Figure S3.** Hub gene analysis. (A) Receiver operating characteristic (ROC) curve analysis of the diagnostic performance of 10 hub genes for Parkinson's disease (PD) in the integrated Gene Expression Omnibus (GEO) datasets (the area under the ROC curve [AUC] values labeled for each gene). (B) BRCA1 immunohistochemistry in mouse substantia nigra (SN) (scale bar = 100 μm). (C) Quantification of BRCA1‐positive cell number. (D) SMARCA4 immunohistochemistry in mouse SN (scale bar = 100 μm). (E) Quantification of SMARCA4‐positive cell number. *n* = 3 biological replicates per group, data are presented as mean ± standard deviation (SD). One‐way analysis of variance (ANOVA) with Tukey's honestly significant difference (HSD) post‐hoc test and Bonferroni's correction. *p < 0.05, ***p < 0.001 versus saline; ^###^ *p* < 0.001 versus d‐gal; ^∆∆^ *p* < 0.01, ^∆∆∆^ *p* < 0.001 versus 1‐methyl‐4‐phenyl‐1,2,3,6‐tetrahydropyridine (MPTP).
**Figure S4.** Pathology index detection of MN9D cell. (A) Immunofluorescence co‐localization of p21 (green) and α‐synuclein (α‐syn) (red) in MN9D cells, with DAPI used for nuclear staining (scale bar = 50 μm) (B) Quantification of P21 and α‐syn mean fluorescence intensity. (C) Senescence‐Tracker fluorescent probe detection of senescent cells (scale bar = 50 μm). (D) Quantification of relative fluorescence intensity. (E) Reactive oxygen species (ROS) fluorescent probe detection of intracellular ROS (scale bar = 50 μm). (F) Quantification of ROS mean fluorescence intensity. *n* = 3 biological replicates per group; data are presented as mean ± standard deviation (SD). One‐way analysis of variance (ANOVA) with Tukey's honestly significant difference (HSD) post‐hoc test and Bonferroni's correction. *p < 0.05, **p < 0.01, ***p < 0.001 versus control; ^##^ *p* < 0.01, ^###^ *p* < 0.001 versus d‐gal; ^∆∆^ *p* < 0.01, ^∆∆∆^ *p* < 0.001 versus MPP^+^.
**Figure S5.** Chemical structure of potential therapeutic drugs, including fumaric acid, 1,2,3‐benzenetricarboxylic acid, hydroxychloroquine, CHEMBL1709259, fostamatinib, MK‐5108, ropsacitinib, ulinastatin, and onalespib.

**Table S1.** Senescence associated gene.
**Table S2.** The biological function of hub genes.
**Table S3.** Prediction of therapeutic drugs.

## Data Availability

The gene expression datasets analyzed in this study are available in the Gene Expression Online Resource (GEO). All data and material generated or analyzed during this study are included in this published article (and its Supplementary Information files).
