# Supplementary material for: Identification of senescence‐related genes in Parkinson's disease reveals candidate therapeutic targets and pathological processes
Source: Animal Model Exp Med. 2026 Jun 30;9(9):1761–81. doi: 10.1002/ame2.70240 (PMC13394503; doi:10.1002/ame2.70240)
Supplement: Supplementary file 1 [file AME2-9-1761-s002.docx]

Figure S1 Gene chip data information. (A) The gene chip expression profiles of 43 brain tissue samples, including 26 PD patients and 17 healthy patients. (B) PCA analysis. (C) The differential gene volcano map. Red represents upregulated genes, and blue represents downregulated genes.

Figure S2 (A) Body weight curve of mice in each group over 8 weeks, n=10 per group. (B) Immunofluorescence staining of TH in mouse SN (red, DAPI for nuclear staining, scale bar=100 μm). (C) Quantification of TH mean fluorescence intensity, n=3 per group. Data are mean ± SD. One-way ANOVA with Tukey’s HSD post-hoc test and Bonferroni correction. **P* < 0.05, ***P* < 0.01, ****P* < 0.001 vs. Saline; ^#^*P* < 0.05, ^##^*P* < 0.01, ^###^*P* < 0.001 vs. D-gal; ^∆^*P* < 0.05 vs. MPTP.

Figure S3 Hub gene analysis. (A) ROC curve analysis of the diagnostic performance of 10 hub genes for PD in the integrated GEO datasets (AUC values labeled for each gene). (B) BRCA1 immunohistochemistry in mouse SN (scale bar=100 μm). (C) Quantification of BRCA1-positive cell number. (D) SMARCA4 immunohistochemistry in mouse SN (scale bar=100 μm). (E) Quantification of SMARCA4-positive cell number. n=3 biological replicates per group, data are mean ± SD. One-way ANOVA with Tukey’s HSD post-hoc test and Bonferroni correction. **P* < 0.05, ****P* < 0.001 vs. Saline; ^###^*P* < 0.001 vs. D-gal; ^∆∆^*P* < 0.01, ^∆∆∆^*P* < 0.001 vs. MPTP.

Figure S4 Pathology index detection of MN9D cell. (A) Immunofluorescence co-localization of p21 (green) and α-syn (red) in MN9D cells, DAPI for nuclear staining (scale bar=50 μm) (B) Quantification of P21 and α-syn mean fluorescence intensity. (C) Senescence-Tracker™ fluorescent probe detection of senescent cells (scale bar=50 μm). (D) Quantification of relative fluorescence intensity. (E) ROS fluorescent probe detection of intracellular ROS (scale bar=50 μm). (F) Quantification of ROS mean fluorescence intensity. n=3 biological replicates per group, data are mean ± SD. One-way ANOVA with Tukey’s HSD post-hoc test and Bonferroni correction. **P* < 0.05, ***P* < 0.01, ****P* < 0.001 vs. Control; ^##^*P* < 0.01, ^###^*P* < 0.001 vs. D-gal; ^∆∆^*P* < 0.01, ^∆∆∆^*P* < 0.001 vs. MPP^+^.

Figure S5 Chemical structure of potential therapeutic drugs, including fumaric acid, 1,2,3-benzenetricarboxylic acid, hydroxychloroquine, CHEMBL1709259, fostamatinib , MK-5108, ropsacitinib, ulinastatin, and onalespib.
